# Association of genetic polymorphisms in *SOD2*, *SOD3*, *GPX3*, and *GSTT1* with hypertriglyceridemia and low HDL-C level in subjects with high risk of coronary artery disease

**DOI:** 10.7717/peerj.7407

**Published:** 2019-08-01

**Authors:** Nisa Decharatchakul, Chatri Settasatian, Nongnuch Settasatian, Nantarat Komanasin, Upa Kukongviriyapan, Phongsak Intharaphet, Vichai Senthong

**Affiliations:** 1 Biomedical Sciences Program, Graduate School, Khon Kaen University, Khon Kaen, Thailand; 2 Cardiovascular Research Group, Khon Kaen University, Khon Kaen, Thailand; 3 Department of Pathology, Faculty of Medicine, Khon Kaen University, Khon Kaen, Thailand; 4 School of Medical Technology, Faculty of Associated Medical Sciences, Khon Kaen University, Khon Kaen, Thailand; 5 Department of Physiology, Faculty of Medicine, Khon Kaen University, Khon Kaen, Thailand; 6 Queen Sirikit Heart Center of the Northeast, Khon Kaen University, Khon Kaen, Thailand; 7 Department of Internal Medicine, Faculty of Medicine, Khon Kaen University, Khon Kaen, Thailand

**Keywords:** Antioxidant, Glutathione peroxidase, Superoxide dismutase, GlutathioneS-transferase, Atherosclerosis, Oxidative stress, Genetic risk score

## Abstract

**Background:**

Oxidative stress modulates insulin resistant-related atherogenic dyslipidemia: hypertriglyceridemia (HTG) and low high-density lipoprotein cholesterol (HDL-C) level. Gene polymorphisms in superoxide dismutase (*SOD2* and *SOD3*), glutathione peroxidase-3 (*GPX3*), and glutathione S-transferase theta-1 (*GSTT1*) may enable oxidative stress-related lipid abnormalities and severity of coronary atherosclerosis. The present study investigated the associations of antioxidant-related gene polymorphisms with atherogenic dyslipidemia and atherosclerotic severity in subjects with high risk of coronary artery disease (CAD).

**Methods:**

Study population comprises of 396 subjects with high risk of CAD. Gene polymorphisms: *SOD2* rs4880, *SOD3* rs2536512 and rs2855262, *GPX* rs3828599, and *GSTT1* (deletion) were evaluated the associations with HTG, low HDL-C, high TG/HDL-C ratio, and severity of coronary atherosclerosis.

**Results:**

*SOD2* rs4880-CC, *SOD3* rs2536512-AA, rs2855262-CC, and *GPX3* rs3828599-AA, but not *GSTT1*^-/-^ individually increased risk of HTG combined with low HDL-C level. With a combination of five risk-genotypes as a genetic risk score (GRS), GRS ≥ 6 increased risks of low HDL-C, high TG/HDL-C ratio, and HTG combined with low HDL-C, comparing with GRS 0–2 [respective adjusted ORs (95% CI) = 2.70 (1.24–5.85), 3.11 (1.55–6.23), and 5.73 (2.22–14.77)]. Gene polymorphisms, though, were not directly associated with severity of coronary atherosclerosis; high TG/HDL-C ratio was associated with coronary atherosclerotic severity [OR = 2.26 (95% CI [1.17–4.34])].

**Conclusion:**

Combined polymorphisms in antioxidant-related genes increased the risk of dyslipidemia related to atherosclerotic severity, suggesting the combined antioxidant-related gene polymorphisms as predictor of atherogenic dyslipidemia.

## Introduction

Atherosclerosis is the major cause of coronary artery disease (CAD). Atherogenesis is a complex interplay of abnormal plasma lipid level (dyslipidemia), oxidative stress, and dysfunctional endothelial cells. In addition, genetic variations have also been implicated as a potent risk-predictor for CAD. Primary low level of high-density lipoprotein cholesterol (HDL-C), isolated hypertriglyceridemia (HTG), high triglycerides (TG) to HDL-C ratio (TG/HDL-C), as well as a combination of high TG with low HDL-C levels have also emerged as risk factors for cardiovascular events, including CAD (Ahmed et al., 2016; Da Luz et al., 2008; Klempfner et al., 2016; Lee et al., 2017).

Oxidative stress is the stage of redox imbalance; where the oxidant activity, mostly from reactive oxygen species (ROS), overwhelms the defense capacity of the endogenous antioxidant system. Oxidative stress plays a role in endothelial dysfunction and atherosclerotic process (Kattoor et al., 2017). A number of in vitro and population studies have provided strong evidence that oxidative stress is the key mechanism in insulin resistance (IR) (Ding et al., 2016; Maslov et al., 2019; Meigs et al., 2007; Park et al., 2009). IR enables adipocyte lipolysis that increases hepatic in-flux of free fatty acids (FFA), by which it induces hepatic TG synthesis. This leads to increased hepatic secretion of TG-rich very low-density lipoprotein (VLDL) and results in elevated blood TG level (Welty, 2013). Moreover, and in vitro study has reported that high ROS levels down-regulated *APOAI* gene expression and ApoA-I secretion. Because ApoA-I is the major protein component in HDL biosynthesis, therefore oxidative stress-related suppression of ApoA-I also has an effect on lowering HDL-C level (Shavva et al., 2017).

The major antioxidant enzymes that maintain redox homeostasis include superoxide dismutase (SOD), glutathione peroxidase (GPX), and glutathione S-transferase (GST). SOD2 and SOD3, which localize respectively within mitochondrial matrix and to the cell surface, catalyze the dismutation of superoxide (O_2_^•−^) into hydrogen peroxide (H_2_O_2_) (Karlsson, Lindahl & Marklund, 1988; Karnati et al., 2013) whereas GPX3, an antioxidant component in HDL fraction, involved in detoxification of extracellular H_2_O_2_ and lipid hydroperoxides (Chen et al., 2000). Both O_2_^• −^ and H_2_O_2_ are potent ROS found in mitochondria, cytosol, and extracellular compartment. There are reports showing that accumulation of these ROS in mitochondria and influx of extracellular ROS contribute to cellular IR (Fazakerley et al., 2018; Iwakami et al., 2011) which may be suppressed by SOD and GPX through their neutralizing of these ROS. Furthermore, over-expression of *Sod2* in the rat (Boden et al., 2012) and *Sod3* in the mouse (Cui et al., 2014) have prevented high fat diet-induced IR. In addition, induced-expression of GPX3 in human skeletal muscle cells has also inhibited oxidative stress-induced IR (Chung et al., 2009), a condition associated with HTG and low HDL-C level. Glutathione S-transferase theta 1 (GSTT1), a member of cytosolic GSTs, plays a key role in termination of phospholipid oxidation by detoxifying phospholipid hydroperoxide (Hurst et al., 1998). In vitro studies have revealed that 4-hydroperoxy-nonenal (4-HNE), an end product of phospholipid oxidation (Reis & Spickett, 2012), can induce IR in adipocytes, and activation of GST protein expression can prevent 4-HNE-induced IR and lipolysis in adipocytes (Liu et al., 2018).

As genetic variations are the underlying risk factors for almost all complex diseases; we speculated that polymorphisms in these antioxidant genes may modify their gene function that affects their antioxidant activities associated with dyslipidemia and risk for CAD. The *SOD2* c.47C>T polymorphism (Ala16Val, rs4880) has been associated with the risks of CAD (Souiden et al., 2016) and type 2 diabetes mellitus (T2DM) (Vats et al., 2015). The *SOD3* c.172G>A polymorphism (Ala58Thr, rs2536512) has also been associated with a risk of T2DM (Yang, Xie & Jin, 2016) but not hypertension (HT) (Dong et al., 2014). Another *SOD3* polymorphism, g.9892T>C (rs2855262), is localized in 3’untranslated region (3’UTR) (Crocco et al., 2016) and, as a linkage single nucleotide polymorphism (SNP) in *SOD3* haplotype, has been associated with decreased severity of acute-infected lung injury (Arcaroli et al., 2009). The *GPX3* c.87+1494A>G polymorphism (rs3828599), localized in the first intron of this gene, has been associated with essential HT (Hao et al., 2011). Several reports have provided the evident association between *GSTT1* deletion polymorphism (*GSTT1* + or −) and risk of CAD; however, with variable results (Song et al., 2017). *GSTT1* deletion carriers have higher blood levels of TG, VLDL-C, and TG/HDL-C ratio (Maciel et al., 2009; Pinheiro et al., 2013) and lower level of HDL-C (Maciel et al., 2009).

Because five polymorphisms from four genes—*SOD2* rs4880, *SOD3* rs2536512 and rs2855262, *GPX3* rs3828599, and *GSTT1* (deletion)—have previously been reported their association with oxidative stress-related CAD risk factors such as HT and T2DM (Hao et al., 2011; Petrovic & Peterlin, 2014; Vats et al., 2015; Yang, Xie & Jin, 2016), they may implicate in lipid abnormalities and severity of coronary atherosclerosis in individuals with CAD. These gene polymorphisms were therefore selected for the present study. However, most of the previous reports have provided evidence form single gene polymorphisms and demonstrated inconsistent results. For complex diseases, genetic associations provided by single polymorphisms are usually of small magnitude (odds ratios 1.1–1.5) and usually elucidate only 1–8% of the overall disease risk in the population; the combined or additive effect of multiple genetic variants from different loci may account for a greater proportion of the disease risk (Ioannidis, 2003). However, the associations of combined polymorphisms in these genes with lipid abnormalities and atherosclerotic severity have never been studied in subjects with high risk of coronary artery disease (CAD). We hypothesized that subjects who carry a combined risk genotype from five polymorphisms would have a greater risk of having lipid abnormalities and contribute to the severity of coronary atherosclerosis compared to those with combined non-risk genotypes. The present study, therefore, aimed to evaluate the associations of individual and combined *SOD2*, *SOD3, GPX3*, and *GSTT1* gene polymorphisms with lipid abnormalities, particularly HTG and low HDL-C level in subjects with high risk of CAD, and to further investigate the associations of these polymorphisms and lipid abnormalities with the severity of coronary atherosclerosis.

## Materials & Methods

### Study population

The study population were recruited from Thai subjects who attended for CAD investigation at Queen Sirikit Heart Center of the Northeast, Faculty of Medicine, Khon Kaen University, Thailand, between 2008 and 2012. These subjects, indicated as “high-risk subjects”, had signs or symptoms suspecting of CAD (reviewed in Cassar et al., 2009) and/or history of cardiovascular risk factors such as smoking, dyslipidemia, diabetes mellitus, hypertension, or obesity. Participants who had unstable angina, left ventricular hypertrophy, heart failure, cardiomyopathy, arrhythmia, and undergoing percutaneous coronary intervention or stent implantation, as well as those who were diagnosed with renal diseases, liver diseases, cancers, and inflammatory diseases, were excluded from the study. After providing the detail of the study, 396 participants, aged between 30 and 86 years, had voluntarily joined the study and gave written consent forms as well as demographic and risk factor information. Physical examination and blood collection were conducted for these participants, before being referred to the Cardiac Catheterization Unit for coronary angiography. There were 389 subjects who underwent elective, non-urgent coronary angiography in the absence of emergency conditions. Based on coronary angiography, 225 patients who had at least one of the main coronary arteries showing ≥50% luminal stenosis were defined as CAD, whereas 164 subjects having none or with <50% luminal stenosis were categorized as non-CAD (Scanlon et al., 1999).

### Ethics statement

The study protocols, as being performed in human participants, were examined and approved by the Khon Kaen University Ethics Committee for Human Research and were in accordance with the 1964 Helsinki Declaration and its later amendments or comparable ethical standards. The approval certificate for Ethics in Human Research for Experimental studies, HE510414, was obtained.

### Assessment for the severity of coronary atherosclerosis

The severity of coronary atherosclerosis, based on angiographic data, was evaluated using “Gensini score (GS)”, which is correlated with “atherosclerotic plaque burden” (Neeland et al., 2012). GS was calculated as the sum of “individual lesion score”—based on the degree of luminal stenosis, the involved coronary vessel, and its location along the target vessel—according to a previously described method (Yongsakulchai et al., 2016). To evaluate the severity of coronary atherosclerosis, the subjects were categorized into two groups using the median GS, 32 points in this study population, as the cut-off point between two groups being assigned as “absent to mild” and “moderate to severe” coronary atherosclerosis.

### Blood biochemical parameters and demographic data

Venous blood was collected after 12 h fasting; for high-risk group, blood was collected before undergoing coronary angiography. Blood lipid parameters and fasting blood sugar (FBS) were determined using Cobas Integra 400 chemistry autoanalyzer (Roche diagnostic, Switzerland). LDL-C was calculated using the Friedewald equation for samples with TG concentrations <4.5 mmol/L. Serum high-sensitivity C-reactive protein (hs-CRP) was measured using the BN ProSpec® System (Siemens Healthcare Diagnostics Products GmbH, Marburg, Germany). Fasting insulin level was determined by sandwich immunoassay (Cisbio Bioassays, Bedford, MA, USA). The homeostatic model assessment of insulin resistance (HOMA-IR) was calculated by [FBS (mmol/L) × fasting insulin level (µU/mL)]/22.5 (Matthews et al., 1985).

Obesity was identified from body mass index (BMI) of ≥25 kg/m^2^ (WHO, 2000). Diabetes mellitus (DM) was identified by prior physician diagnosis or FBS ≥7.0 mmol/L (American Diabetes Association, 2013). Hypertension (HT) was defined if: (i) subject received the antihypertensive drug, or (ii) from prior physician diagnosis, or (iii) whose blood pressure reading ≥140 mmHg for systolic blood pressure (SBP) or ≥90 mmHg for diastolic blood pressure (DBP) (Weber et al., 2014). All abnormal lipid and lipoprotein profiles were followed the National Cholesterol Education Program and Adult Treatment Panel III (NCEP-ATP III) guideline. Low HDL-C level was considered from a value of HDL-C below the 50th percentile (HDL-C <1.0 mmol/L). Hypertriglyceridemia (HTG) was defined by fasting serum for TG level of ≥1.7 mmol/L. Subject who had an abnormal level of one or more lipid profiles, or currently on the lipid-lowering drug, or had a history of lipid disorder was defined as “dyslipidemia (DL)”. The cut-off point for “high TG/HDL ratio (in mg/dL unit)” in the present study was 3.7, as determined by constructing the ROC curves for prediction of atherosclerotic severity. In addition, the TG/HDL-C ratio ≥3, as a surrogate marker of insulin resistance (IR) (Hannon et al., 2006; Iwani et al., 2017) was also included in the evaluation. Metabolic syndrome (MetS) was defined for subjects who had three or more of five conditions according to the International Diabetes Federation (IDF) and NCEP-ATPIII criteria.

### Genotyping of antioxidant gene polymorphisms

The polymorphisms of antioxidant genes were selected based on data from the previous reports showing evidence of association with CVD risk factors in various populations, except for *SOD3* g.9892T>C (rs2855262). This SNP comprises two polymorphic, T and C alleles that generate three possible genotypes (TT, TC, and CC). Although there was no report of association between *SOD3* rs2855262 and CVD risk factors, this SNP was selected based on prior pilot study showing its relationship with oxidative stress markers in subjects with MetS, aged >50 years; subjects with CC had a higher level of malondialdehyde (MDA) than those with TT (*p* = 0.010) (Table S1).

Genomic DNA was extracted from peripheral blood leukocytes using the Flexi Gene DNA isolation kit (QIAGEN, Germany). *GSTT1* deletion polymorphism was determined by multiplex polymerase chain reaction (multiplex PCR) previously described by Sprenger et al. (2000) with some modification. *SOD2, SOD3,* and *GPX3* SNPs were determined using allele-specific polymerase chain reaction (AS-PCR) technique. GeneFisher software (Giegerich, Meyer & Schleiermacher, 1996) was used in designing the PCR primers. Details of genotyping are provided in the Data S1.

For each gene polymorphism, the genotyping results were validated by DNA sequencing (1st BASE, Selangor, Malaysia) of at least 10% randomly selected samples. The DNA sequencing results confirmed all genotyping results of all gene polymorphisms in all randomly selected samples (examples of DNA sequencing profiles are shown in Data S2).

### Genetic risk scores

To evaluate the combined effect, genetic risk score (GRS) was formulated based on risk-contributing alleles across multiple polymorphisms. For each bi-allelic locus, an individual may possibly have risk score 0, 1, or 2. A GRS was calculated by the summation of risk alleles from all five polymorphisms: *SOD2* rs4880, *SOD3* rs2536512, *SOD3* rs2855262, *GPX3* rs3828599, and *GSTT1* (deletion). For five polymorphisms, an individual would theoretically have GRS between scores 0 and 10.

### Statistical analysis

Statistical analysis was performed using the SPSS software version 17 (SPSS inc., Chicago, IL). The genotype frequency and categorical variable differences between case and control subjects were assessed by Chi-square analysis. The genotype distributions were tested for Hardy–Weinberg equilibrium (HWE) using HW calculator provided by Santiago Rodriguez (http://www.oege.org/software/hwe-mr-calc.shtml). The associations between genotypes and the risks of metabolic disorders were analyzed using a logistic regression model and expressed as Odds Ratio (OR) with 95% confidence interval (95% CI). For multiple testing, the results for the GRS-phenotype associations were corrected using a Bonferroni correction, based on the number of calculated GRSs (*n* = 5). This study set significance at a Bonferroni corrected alpha (*α*) [corrected *p* < 0.010, *α* = 0.050∕5]. Continuous variables were first tested for normal distribution utilizing the Kolmogorov–Smirnov normality test. Those variables with skewed distribution were log_10_-transformed before subsequent data analysis. Student’s *t*-test was used to compare the means of continuous variables between two groups. One way analysis of variance (ANOVA) was used to compare variables among three or more groups and followed by a *post hoc* test (Scheffe’s procedure). The polynomial linear trend test was used to assess linear trends across genotype or GRS groups for continuous variables. All continuous variables were expressed as means ± standard deviation (SD). Statistical significance was considered as a two-tailed *p*-value <0.05. Sample size and power calculations are shown in Table S2.

## Results

### Baseline demographics and clinical characteristics of the study population

For further analysis, the study population was first divided into two groups based on the degree of coronary atherosclerosis, using Gensini score (GS) 32 as the cut-off point, and was assigned as “absent to mild” (GS ≤ 32) and “moderate to severe” (GS > 32) coronary atherosclerosis. The baseline demographics and clinical characteristics of study subjects based on GS are demonstrated in Table 1. The number of male subjects in “moderate to severe” coronary atherosclerosis group (70.2%) was higher than in “absent to mild” coronary atherosclerosis group (53.4%), *p* = 0.002. The frequencies of subjects with high TG/HDL-C ratio were significantly higher in “moderate to severe” than in “absent to mild” groups. As compared with “absent to mild”, patients with “moderate to severe” atherosclerosis had significantly higher average age, Gensini score (GS), and waist/hip ratio; higher levels of TC, TG, LDL-C, and TG/HDL-C; and lower level of HDL-C.

**Table 1:** Baseline demographics and clinical characteristics of the study population.

**Parameter**	**Gensini score≤32; *N* (%)(*n* = 268)**	**Gensini score>32; *N* (%)(*n* = 121)**	***p*-value**
Gender:			
Male, *n* (%)	143 (53.4%)	85 (70.2%)	**0.002**
Female, *n* (%)	125 (46.6%)	36 (29.8%)	
Smoking status; *n* (%)	89 (33.2%)	51 (42.1%)	0.089
High TG/HDL-C (≥3; unit mg/dL)	158 (59.0%)	87 (71.9%)	**0.014**
High TG/HDL-C (≥3.7; unit mg/dL)	124 (46.3%)	72 (59.5%)	**0.016**
Metabolic syndrome; *n* (%)	154 (60.4%)	78 (67.8%)	0.171
CAD; *n* (%)	105 (39.2%)	120 (99.2%)	**<0.001**
Age (year)	60.2 ± 9.0	63.1 ± 9.2	**0.004**
Gensini score	7.1 ± 9.7	67.7 ± 33.2	**<0.001**
HOMA-IR	5.29 ± 5.09	7.47 ± 12.23	0.102
SBP (mmHg)	129.5 ± 18.7	129.4 ± 18.5	0.972
DBP (mmHg)	74.1 ± 10.3	72.5 ± 11.6	0.118
BMI (kg/m^2^)	24.7 ± 3.7	24.7 ± 3.0	0.680
Waist/hip ratio	0.94 ± 0.07	0.97 ± 0.07	**0.014**
FBS (mmol/L)	6.21 ± 2.61	6.44 ± 2.66	0.395
TC (mmol/L)	4.43 ± 1.08	4.75 ± 1.46	**0.033**
TG (mmol/L)	1.86 ± 1.05	2.18 ± 1.31	**0.013**
HDL-C (mmol/L)	1.10 ± 0.34	1.00 ± 0.26	**0.009**
LDL-C (mmol/L)	2.48 ± 0.91	2.77 ± 1.16	**0.017**
TG/HDL-C (unit: mmol/L)	1.95 ± 1.54	2.46 ± 1.86	**0.003**
TG/HDL-C (unit: mg/dL)	4.46 ± 3.53	5.62 ± 4.25	**0.003**
hsCRP (mg/L)	4.10 ± 10.16	4.11 ± 7.01	0.492

**Notes.**

Abbreviations HOMA-IR Homeostatic Model Assessment for Insulin Resistance SBP systolic blood pressure DBP diastolic blood pressure BMI body mass index FBS fasting blood sugar TC total cholesterol TG triglycerides HDL-C high density lipoprotein cholesterol LDL-C low density lipoprotein cholesterol hs-CRP high-sensitivity C-reactive protein

Categorical variables are presented as percentage of subject in each group and continuous variables are presented as mean ± SD.

### Associations of *SOD2*, *SOD3*, *GPX3*, and *GSTT1* polymorphisms with TG and HDL-C dyslipidemia

The distribution of genotypes and alleles for all gene polymorphisms in the study population followed HWE (*p* > 0.05). The frequencies of minor alleles: *SOD2* rs4880-C, *SOD3* rs2536512-A, *SOD3* rs2855262-C, *GPX3* rs3828599-A, and *GSTT1* (+) were 25.0%, 39.1%, 42.7%, 43.3%, and 42.7%, respectively. Linkage disequilibrium (*D*′ = 0.82) between two *SOD3* polymorphisms was also observed in this population. The step-wise relationship between genotypes and TG and HDL-C levels in each individual polymorphism is summarized in Table 2 (showing *p* for trend)**.**

**Table 2:** The relationship between genotypes/GRS groups and TG and HDL-C levels for each individual polymorphism and for a combined risk-alleles (GRS).

**Polymorphisms**		**TG(mmol/L)**	**HDL-C(mmol/L)**	**TG/HDL-C(unit: mmol/L)**	**TG/HDL-C(unit: mg/dL)**
*SOD2* rs4880	TT (*n* = 223)	1.83 ± 0.93	1.06 ± 0.33	1.99 ± 1.40	4.57 ± 3.20
	TC (*n* = 148)	2.07 ± 1.39	1.11 ± 0.34	2.16 ± 2.02	4.95 ± 4.63
	CC (*n* = 25)	2.45 ± 1.56	0.99 ± 0.25	2.82 ± 2.24	6.46 ± 5.12
	*p*-value	0.049	0.137	0.102	0.102
	*p* for trend^*^	0.020	0.330	0.033	0.033
*SOD3* rs2536512	GG (*n* = 146)	1.82 ± 1.10^a^	1.10 ± 0.33	1.88 ± 1.52^b^	4.30 ± 3.47^b^
	GA (*n* = 190)	1.98 ± 1.24	1.09 ± 0.34	2.15 ± 1.87	4.92 ± 4.28
	AA (*n* = 60)	2.22 ± 1.09^a^	0.99 ± 0.28	2.54 ± 1.63^b^	5.82 ± 3.74^b^
	*p*-value	0.037	0.052	0.014	0.014
	*p* for trend^*^	0.010	0.016	0.004	0.004
*SOD3* rs2855262	TT (*n* = 136)	1.92 ± 1.36	1.12 ± 0.36^c^	2.04 ± 2.05^d^	4.66 ± 4.69^d^
	TC (*n* = 182)	1.93 ± 1.10	1.08 ± 0.32	2.04 ± 1.50	4.67 ± 3.44
	CC (*n* = 78)	2.08 ± 0.98	0.99 ± 0.28^c^	2.39 ± 1.56^d^	5.48 ± 3.57^d^
	*p*-value	0.133	0.020	0.029	0.029
	*p* for trend^*^	0.047	0.006	0.009	0.009
*GPX3* rs3828599	GG (*n* = 127)	1.96 ± 1.22	1.13 ± 0.36	2.09 ± 1.81	4.79 ± 4.16
	GA (*n* = 195)	1.95 ± 1.23	1.06 ± 0.32	2.14 ± 1.85	4.89 ± 4.25
	AA (*n* = 74)	1.97 ± 0.91	1.05 ± 0.29	2.06 ± 1.09	4.73 ± 2.51
	*p*-value	0.675	0.158	0.483	0.483
	*p* for trend^*^	0.416	0.175	0.243	0.243
*GSTT1* gene deletion polymorphism	+∕ + (*n* = 79)	1.90 ± 1.02	1.11 ± 0.35	1.96 ± 1.31	4.48 ± 3.00
	+∕ − (*n* = 180)	1.98 ± 1.25	1.09 ± 0.34	2.14 ± 1.85	4.91 ± 4.23
	−∕ − (*n* = 137)	1.95 ± 1.16	1.04 ± 0.30	2.15 ± 1.76	4.92 ± 4.04
	*p*-value	0.980	0.337	0.806	0.806
	*p* for trend^*^	0.901	0.190	0.525	0.525
Genetic risk score (GRS)	0–2 (*n* = 73)	1.72 ± 0.96	1.16 ± 0.36^e^	1.74 ± 1.30^f^	3.97 ± 2.97^f^
	3 (*n* = 82)	1.92 ± 1.22	1.10 ± 0.36	2.06 ± 1.78	4.73 ± 4.07
	4–5 (*n* = 155)	1.98 ± 1.24	1.07 ± 0.31	2.15 ± 1.89	4.93 ± 4.33
	6–10 (*n* = 86)	2.13 ± 1.16	1.00 ± 0.28^e^	2.38 ± 1.63^f^	5.46 ± 3.73^f^
	*p*-value	0.092	0.041	0.023	0.023
	*p* for trend^*^	0.011	0.004	0.002	0.002

**Notes.**

Continuous variables are presented as mean ± SD.

* By ANOVA with polynomial contrasts for linear trend.

a *p*-value = 0.037.

b *p*-value = 0.014.

c *p*-value = 0.022.

d *p*-value = 0.032.

e *p*-value = 0.046.

f *p*-value = 0.026.

**Figure 1:**
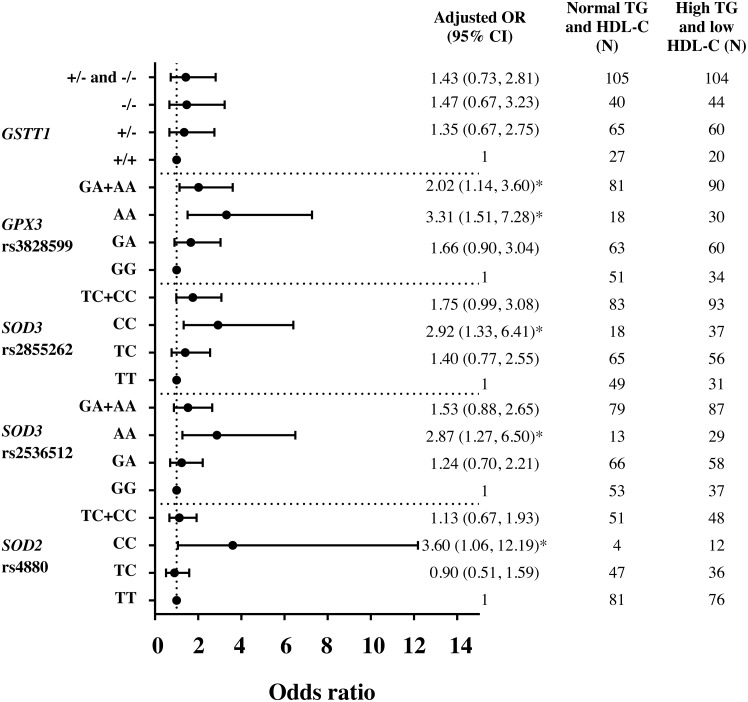
Adjusted Odds ratios (ORs) and 95% CI for the risk of HTG and low HDL-C (<1 mmol/L) as the function of antioxidant related gene polymorphisms. The ORs were adjusted for age, gender, smoking status, and administration of fibrate. N, total number of genotype carriers in each group. * indicates statistically significant; *p* < 0.05.

Assessment by logistic regression for *SOD* 2 c.47C>T (rs4880), CC genotype significantly increased the risks of HTG [adjusted OR = 3.23 (95% CI [1.20–8.75]), *p* = 0.021 (Fig. S1)], high TG/HDL-C ratio (≥3.7) [adjusted OR = 2.57 (95% CI [1.06–6.24]), *p* = 0.037 (Fig. S2)] and HTG combined with low HDL-C (<1 mmol/L) [adjusted OR = 3.60 (95% CI [1.06–12.19]), *p* = 0.040 (Fig. 1)], as compared with TT. With linear regression analysis, significant associations of TC and CC genotypes with TG (log-transformed) levels were also revealed as compared with TT genotype, adjusted for age, gender, HDL-C level, smoking status, and administration of fibrate (*β* = 0.046 *p* = 0.035 and *β* = 0.098 *p* = 0.023). Moreover, CC genotype was significantly associated with TG/HDL-C ratio (log-transformed), adjusted for age, gender, smoking status, and administration of fibrate, *β* = 0.169, *p* = 0.009.

For *SOD3* c.172G>A (rs2536512), comparing with GG genotype, AA genotype had higher levels of TG and TG/HDL-C ratio (Table 2) and significantly associated with HTG [adjusted OR = 2.09 (95% CI [1.08–4.05]), *p* = 0.029 (Fig. S1)]. Moreover, AA carriers were also associated with risk of having HTG combined with low HDL-C (<1 mmol/L) as compared with GG [adjusted OR = 2.87 (95% CI [1.27–6.50]), *p* = 0.011 (Fig. 1)]. After adjusting for confounding factors, a significant association of AA carriers with TG/HDL-C ratio (log-transformed) levels was also presented as compared with GG carriers (*β* = 0.111, *p* = 0.015).

*SOD3* g.9892T>C (rs2855262), compared with TT, CC carriers had lower HDL-C level and higher TG/HDL-C ratio (Table 2) and were associated with risk of having HTG combined with low HDL-C [adjusted OR = 2.92 (95% CI [1.33–6.41]), *p* = 0.007 (Fig. 1)]. Furthermore, CC genotype was also significantly associated with TG/HDL-C ratio (log-transformed) after adjusting for confounding factors, *β* = 0.096, *p* = 0.023.

Both *GPX3* c.87+1494A>G (rs3828599) and *GSTT1* deletion polymorphisms revealed no genotype relationship with abnormal lipid level (Table 2). When assessed by regression analysis, however, individuals with *GPX3* rs3828599 AA genotype, as comparing with GG carriers, increased risks of high TG/HDL-C ratio [adjusted OR = 2.48 (95% CI [1.34–4.59]), *p* = 0.004 (Fig. S2)], having low HDL-C level [<1 mmol/L; adjusted OR = 2.22 (95% CI [1.17–4.20]), *p* = 0.014 (Fig. S3)], and HTG combined with low HDL-C [adjusted OR = 3.31(95% CI [1.51–7.28]), *p* = 0.003 (Fig. 1)]. There were no associations of *GSTT1* deletion polymorphisms with either HTG alone or HTG combined with low HDL-C level; however, *GSTT1*^-/-^ carriers revealed marginal association with low HDL-C level as compared with *GSTT1*^+/+^ genotype [adjusted OR = 1.70 (95% CI [0.92–3.15]), *p* = 0.092 (Fig. S3)]. In addition, comparing between HOMA-IR ≥5.75 (tertile 3rd) and ≤3.40 (tertile 1), *GSTT1*^-/-^ carriers were associated with high HOMA-IR as compared with *GSTT1* presence (*GSTT1*^+/+^) carriers; OR = 2.29 (95% CI [1.09–4.82]), *p* = 0.028 (adjusted for age, gender, DM, HTG, obesity, and cigarette smoking).

The “power of test” was also determined. Overall, this study has more than 80% power of determining for all alleles that carried OR of 2 or more (*p* < 0.05 as shown in Table S2).

### Effect of combined *SOD2*, *SOD3*, *GPX3*, and *GSTT1* polymorphisms on the risk of TG and HDL-C dyslipidemia

**Figure 2:**
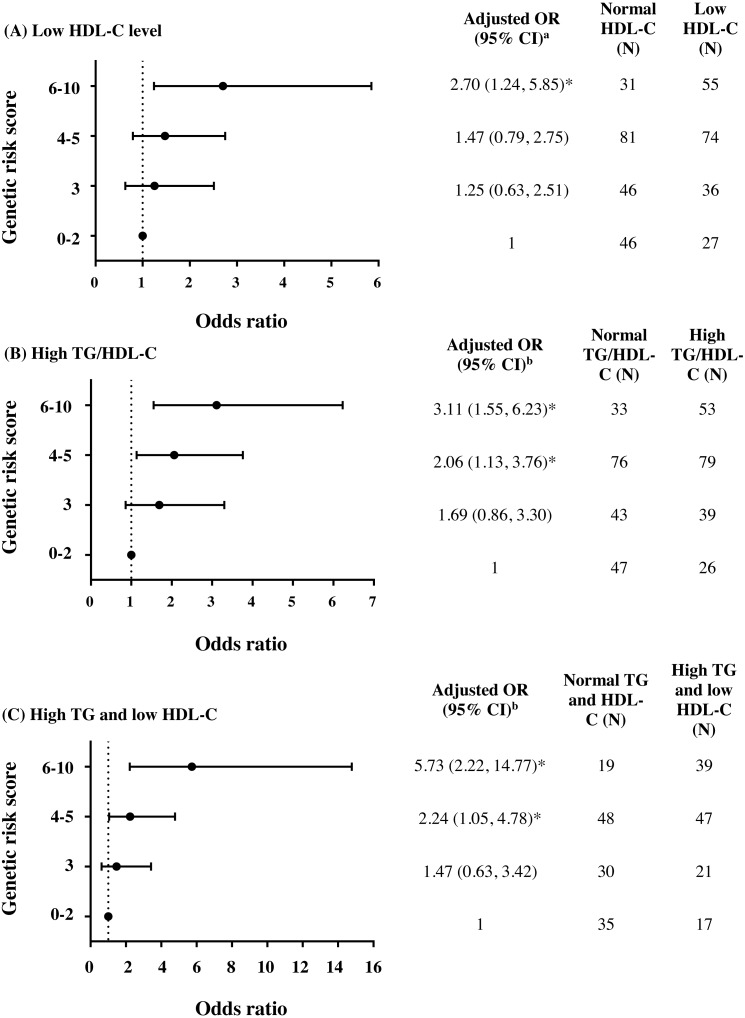
Adjusted ORs and 95% CI for the risks of low HDL-C level, high TG/HDL-C ratio (≥3.7), and HTG combined with low HDL-C as the function of genetic risk score. The adjusted ORs indicating the function of combined five polymorphisms by using genetic risk score 0–2, 3, 4–5, and 6–10 are presented in (A) for low HDL-C level, (B) for high TG/HDL-C ratio, and (C) for HTG combined with low HDL-C. ^a^ The ORs were adjusted for age, gender, prevalence of HTG, smoking status, and administration of fibrate and ^b^ adjusted for age, gender, smoking status, and administration of fibrate. *N*, total number of combined genotype carriers in each group. * indicates statistically significant; *p* < 0.05.

To further investigate the combined effect of these five antioxidant gene polymorphisms, the genetic risk score (GRS) was constructed based on the sum of risk alleles in individual subjects. GRS was formulated based on the risk-contributing alleles across multiple polymorphisms that were significantly associated with HTG, low HDL-C, high TG/HDL-C ratio, combined HTG and low HDL-C, and insulin resistance (high HOMA-IR) in this study**.** Subjects were first categorized into four GRS groups, defined by the quartiles of all subjects, as having GRSs 0–2 (*N* = 73), 3 (*N* = 82), 4–5 (*N* = 155), and 6–10 (*N* = 86). The GRS 0–2 was then used as a reference for risk assessment in other higher GRS groups. As expected, high GRS revealed a significant association with lipid abnormalities. The group of GRS 6-10 had lower HDL-C level and higher TG/HDL-C ratio (Table 2) and presented with increased risk of having low HDL-C level (Fig. 2A), high TG/HDL-C ratio (Fig. 2B), and HTG combined with low HDL-C level (Fig. 2C) as compared with group of GRS 0–2 [respective adjusted ORs (95% CI) = 2.70 (1.24–5.85), *p* = 0.012; 3.11 (1.55–6.23), *p* = 0.001; and 5.73 (2.22–14.77), *p* < 0.001]. The associations of GRS with lipid abnormalities, except low HDL-C, remained statistically significant after applying Bonferroni correction (*p* < 0.010). The associations of GRS 4–5 and GRS 6–10 with TG/HDL-C ratio (log-transformed) were also significant, as compared with GRS 0–2, after adjusting for confounding factors (*β* = 0.091, *p* = 0.030 and *β* = 0.150, *p* = 0.002).

### Associations of TG and HDL-C dyslipidemia with the severity of coronary atherosclerosis

To evaluate if such abnormalities of TG and HDL-C levels could predict the severity of coronary atherosclerosis, association studies were conducted for atherosclerotic severity (Gensini score >32) as a function of lipid abnormalities as well as other CVD risk factors. Only high TG/HDL-C ratio (≥3.7) was associated with severity of coronary atherosclerosis (GS >32) with adjusted OR = 2.26 (95% CI [1.17–4.34]) (Table 3). Furthermore, by using a number of vessels with ≥50% stenosis as an indicator of atherosclerotic severity, high TG/HDL-C ratio (≥3.7) was also associated with multi-vessel disease (≥2) as compared with 0-1 vessel with stenosis, adjusted OR = 2.18 (95% CI [1.20–3.99]).

**Table 3:** The associations of CVD risk factors with the severity of coronary atherosclerosis.

**CVD risk factors**	**Gensiniscore≤32; *N* (%)(*n* = 268)**	**Gensini score>32; *N* (%)(*n* = 121)**	**CrudeOdds Ratio (95%CI)**	***p*-value**	**AdjustedOdds Ratio(95% CI)**	***p*-value**
Diabetes mellitus (DM)	79 (29.6%)	46 (38.7%)	1.50 (0.95, 2.36)	0.080	1.22 (0.62, 2.40)^a^	0.559
Hypertension (HT)	220 (82.1%)	106 (87.6%)	1.54 (0.83, 2.88)	0.174	1.12 (0.42, 3.01)^b^	0.821
Dyslipidemia (DL)	263 (98.1%)	119 (98.3%)	1.13 (0.22, 5.91)	0.884	0.68 (0.10, 4.58)^c^	0.695
Obesity (OB)	115 (42.9%)	50 (42.0%)	0.96 (0.62, 1.49)	0.870	0.96 (0.61, 1.52)^d^	0.877
Hypertriglyceridemia (HTG)	127 (47.4%)	68 (56.2%)	1.42 (0.92, 2.19)	0.108	1.13 (0.60, 2.13)^c^	0.700
Low HDL-C level	128 (47.8%)	63 (52.1%)	1.19 (0.77, 1.83)	0.432	1.27 (0.67, 2.41)^c^	0.461
High TG/HDL-C (≥3)	158 (59.0%)	87 (71.9%)	1.78 (1.12, 2.84)	**0.015**	1.95 (0.95, 4.00)^c^	0.069
High TG/HDL-C (≥3.7)	124 (46.3%)	72 (59.5%)	1.71 (1.10, 2.64)	**0.016**	2.26 (1.17, 4.34)^c^	**0.015**
HTG with normal HDL-C level	47 (17.5%)	25 (20.7%)	1.50 (0.80, 2.81)	0.206	0.82 (0.29, 2.33)^c^	0.705
Low HDL-C level with normal TG level	48 (17.9%)	20 (16.5%)	1.17 (0.61, 2.26)	0.631	1.17 (0.44, 3.09)^c^	0.750
HTG and low HDL-C level	80 (29.9%)	43 (35.5%)	1.51 (0.88, 2.61)	0.134	1.36 (0.57, 3.25)^c^	0.485

**Notes.**

a Adjusted for age, gender, HT, waist/hip ratio, and cigarette smoking.

b Adjusted for age, gender, DM, waist/hip ratio, and cigarette smoking.

c Adjusted for age, gender, HT, DM, waist/hip ratio, and cigarette smoking.

d Adjusted for age, gender, HT, DM, and cigarette smoking.

### Genetic polymorphisms in *SOD2*, *SOD3*, *GPX3*, and *GSTT1* and severity of coronary atherosclerosis

Further investigation was carried out for the possibility that risk genotypes in these antioxidant genes could be also associated with the severity of atherosclerosis. There were no associations of all gene variants with the severity of coronary atherosclerosis (GS >32, Table S3). However, *GSTT1* deletion polymorphism appeared to have the relationship with step-wise increasing GS in all subjects; the mean level of GS for each genotype was 21.5 ± 32.7 for +∕ + (*N* = 75), 26.9 ± 35.4 for +/ − (*N* = 178), and 27.1 ± 34.5 for −/− (*N* = 136), *p* = 0.120, *p* for trend = 0.041. Although there were no significant associations of GRS with the severity of coronary atherosclerosis (Table S4), *GSTT1*^-/-^ carriers were associated with increased risk of multi-vessel CAD (≥2 vessels with ≥50% stenosis) as compared with *GSTT1*^+/+^ carriers (OR = 2.23, 95% CI [1.10–4.51], *p* = 0.026, adjusted for age, gender, DM, HT, waist/hip ratio, and cigarette smoking).

## Discussion

The present study explored the association of five antioxidant-related gene variants with atherogenic dyslipidemia in subjects with high-risk of CAD. From five gene variants being tested, only polymorphisms in *SOD2, SOD3* and *GPX3* were significantly associated with HTG combined with low HDL-C (Fig. 1). For *SOD2* c.47C>T (rs4880), CC genotype significantly increased risks of HTG (Fig. S1) and high TG/HDL-C ratio (Fig. S2), as compared with TT. *SOD3* c.172G>A (rs2536512) significantly associated with HTG, whereas *GPX3* c.87+1494A>G (rs3828599) revealed association with risks of having low HDL-C level (Fig. S3) and high TG/HDL-C. *GSTT1*^-/-^ carriers, though did not have a significant association with such abnormal lipid levels, were associated with increased risk of high IR state (high HOMA-IR), a condition related to TG and HDL-C dyslipidemia. Further combined effect of five polymorphisms, expressed as a genetic risk score (GRS), was investigated; the high GRS pronounced significant association with low HDL-C, high TG/HDL-C ratio, and HTG combined with low HDL-C; with stronger effect (higher OR) was observed for both high TG/HDL-C ratio and HTG combined with low HDL-C, as compared with the effect of individual polymorphisms (Fig. 2) (significant association remained after Bonferroni correction), suggesting the interaction or additive effect of these polymorphisms on such atherogenic lipid abnormality. In the present study, the patients with “moderate to severe” atherosclerosis had significantly higher waist/hip ratio (WHR) as compared with control groups and denoted as abdominal obesity, defined by mean level of WHR above 0.95 for males and above 0.80 for females (Lean, Han & Morrison, 1995). The previous studies have reported significant positive relationships of WHR to the presence of CAD (Rashiti, Behluli & Bytyqi, 2017) and atherosclerotic severity (Scicali et al., 2018), indicating abdominal obesity as an independent risk factor for CAD. We also demonstrated that high TG/HDL-C ratio (≥3.7) independently associated with severity of coronary atherosclerosis, by using either GS >32 or the number of vessels with 50% stenosis as an indicator. This finding may indicate such a high TG/HDL-C ratio as a potent predictor for the severity of coronary atherosclerosis. Although a combination of five antioxidant-related gene variants was not directly associated with severity of coronary atherosclerosis; they were, however, strongly associated with combined HTG and low HDL-C as well as high TG/HDL-C ratio (≥3.7), atherogenic dyslipidemia showing association with atherosclerotic severity. To the best of our knowledge, the present study is the first report for the combined effect of five genetic variants in these antioxidant-related genes on lipid abnormalities associated with severity of coronary atherosclerosis in subjects with high risk of CAD.

Regarding TG and HDL-C dyslipidemia, high TG/HDL-C ratio has also been reported with increased risks of atherosclerosis and arterial stiffness in patients with diabetes (Shimizu et al., 2013), and has been a strong independent predictor of CVD mortality, as well as incident T2DM (Vega et al., 2014). A combination of high TG with low HDL-C levels has also recently been reported to increase risks of both CAD and stroke, particularly in subjects with diabetes (Lee et al., 2017). High TG/HDL-C ratio has been associated with the extent of coronary disease and used as a surrogate marker for insulin resistance (IR) (Da Luz et al., 2008; Iwani et al., 2017).

The prevalence of subjects with high TG/HDL-C ratio [ratio ≥3 (Hannon et al., 2006; Iwani et al., 2017; McLaughlin et al., 2003)] was high, and almost all subjects (95.3%) had high HOMA-IR [≥1.56 for men and ≥1.64 for women (Do et al., 2010)], indicating the presence of IR state. HTG is the most common lipid abnormality in IR state; is due to increased production of TG-rich lipoprotein (VLDL), and is related to the occurrences of small dense LDL particles and low HDL-C level which play mechanistically role in atherogenesis and atherosclerotic progression (Welty, 2013). Therefore, lipid abnormalities in IR condition could pronounce the effect toward increasing severity of atherosclerosis, as seen in the present report.

There was an indirect genotype-outcome relationship regarding *SOD* gene variants associated with TG and HDL-C dyslipidemia. *SOD2* rs4880 causes Ala (GCT) to Val (GTT) substitution in mitochondrial targeting peptide. The Ala-SOD2 can efficiently import into the mitochondrial matrix, whereas the Val variant causes the partial arrest of the precursor within the inner membrane and reduced the formation of active SOD2, resulting in lower SOD2 activity (Fujimoto et al., 2008; Sutton et al., 2003). However, in the present study, *SOD2* rs4880-CC genotype (Ala-SOD2) increased the risks of having HTG and HTG combined with low HDL-C levels. Under metabolic disorder, higher SOD2 activity in subjects with rs4880-CC might cause higher mitochondrial H_2_O_2_ accumulation, leading to oxidative stress-related IR which affect HTG and low HDL-C level (Li et al., 2014). In accordance with animal studies, high-fat diet (HFD)-induced mitochondrial O_2_^• −^ overproduction in mouse adipocytes has resulted in increasing the rate of conversion into H_2_O_2_ by SOD2 and involved in IR-related HTG (Fisher-Wellman & Neufer, 2012; Li et al., 2014). Adipocyte-targeted *Sod2* knockout mice prevent HFD-induced obesity and IR via increasing in mitochondrial biogenesis and fatty acid oxidation, leading to the clearance of circulating FFA and the improvement of IR (Han et al., 2016).

In a previous report, individuals with *SOD3* rs2536512-A allele, causing Ala to Thr substitution at amino acid position 58 in SOD3, has presented with higher SOD activity compared to those with G allele (Dong et al., 2014). In the present study, *SOD3* rs2536512-AA genotype increased the risks of having HTG and HTG combined with low HDL-C levels. In accordance with our finding, a previous study has also demonstrated that *SOD3* rs2536512-A allele carriers have higher IR and an increased risk of T2DM (Tamai et al., 2006). Furthermore, other studies have reported that serum SOD level and SOD activity are negatively correlated with HDL-C and positively correlated with TG levels in patients with cardiovascular risk factors (Gomez-Marcos et al., 2016; Yubero-Serrano et al., 2013). High SOD3 activity was expected to effectively neutralize excess O_2_^• −^ level, however it could result in overproduction of H_2_O_2_, which could accumulate and induce hepatic IR (Iwakami et al., 2011). IR state promotes hepatic VLDL overproduction (increasing de novo lipogenesis) and VLDL secretion, and causes delayed clearance of plasma TG-rich lipoproteins (Taskinen & Boren, 2015). This results in elevated blood TG level which affects HDL level and particle size. High level of TG-rich lipoproteins induces cholesteryl ester transfer protein (CETP) activity in catalyzing the transfer of cholesteryl ester (CE) from HDL to TG-rich lipoproteins in exchange for TG. This exchange results in the small (TG-rich and CE-poor) HDL particles which are catabolized faster than large (CE-rich) HDL, resulting in lower levels of HDL-C (Welty, 2013). For *SOD3* rs2855262, in our pilot study, the CC carriers in older subjects with MetS had the highest level of MDA, an oxidative marker, compared with other genotypes (Table S1). Furthermore, the *SOD3* rs2855262 had strong linkage disequilibrium with *SOD3* rs2536512. Consequently, the association between this SNP and HTG combined with low HDL-C level were also observed.

Regarding *GPX3* rs3828599, by which AA carrier was also strongly associated with low HDL-C level and HTG combined with low HDL-C. Functionally, the AA genotype has been reported with increasing level of *GPX3* expression (Wang et al., 2010). In animal study, human *GPX3* transgenic mice had lower levels of TC and HDL-C compared to wild-type mice (Yang et al., 2009). These may suggest that high GPX3 activity promote such lipid abnormalities. Because GPX3 function in detoxifying H_2_O_2_ requires reduced glutathione (GSH), high GPX3 activity in the presence of high H_2_O_2_ level may lead to the reduction of GSH level. Majority of our study subjects had advanced age and a high prevalence of DM and MetS. GSH levels decline with age (Maher, 2005) and decrease in an individual with DM and MetS (Spanidis et al., 2016). Low GSH redox activity plays a major role in reducing insulin sensitivity (De Mattia et al., 1998), which could contribute to HTG and low HDL-C. In addition, GSH indirectly increases HDL-mediated cholesterol efflux from macrophages via ABCA1 and ABCG1 transporters (Rosenblat et al., 2014); and GSH-treated macrophage expresses a higher level of *PPAR α* and *ABCA1* mRNA than in control cells (Rosenblat et al., 2014). GSH may, therefore, be the mediator influencing both TG and HDL levels.

Altogether, the association of *SOD2*, *SOD3* and *GPX3* polymorphisms with atherogenic dyslipidemia, observed in the present study, could result from the interplay of gene polymorphisms, their modified protein functions, and their involved antioxidant pathway, as well as the underlying condition of the study population. However, further investigation is required to confirm this hypothesis.

The association between *GSTT1* deletion polymorphism and CAD risk has remained controversial (Du et al., 2012). In the present study, *GSTT*^-/-^ had a marginal association with severity of atherosclerosis and low HDL-C level; however, *GSTT*^-/-^ carriers were associated high HOMAR-IR, a state of IR that may contribute to HTG and low HDL-C dyslipidemia in combination with other gene polymorphisms. GSTT1, a cytosolic GST, plays an important role in detoxifying lipid peroxidation products (Hurst et al., 1998). In animal studies, those with lower GST activity had higher levels of TC, TG, phospholipids and lipid peroxidation (Suzumura et al., 2000). In human studies, *GSTT*^-/-^ carriers in adults had a lower level of HDL-C and a higher level of TG/HDL-C ratio (Maciel et al., 2009). However, the molecular mechanism underlying this association remains unclear.

A previous study in the Hungarian population has reported the relationship between catalase gene *(CAT*) polymorphism (C-262T, rs1001179) and HDL-C levels (Goth et al., 2012); T2DM patients with TT had significantly lower HDL-C level than CC carriers. The present study, however, did not explore this SNP in the Thai population because rare minor allele frequency (4%) has been reported in the Asian population (Liu et al., 2016).

The limitation of this study was the absence of functional studies to investigate the impact of the different genotypes on antioxidant gene expression and/or enzyme activity as well as oxidative stress markers in question because of an insufficient amount of fresh blood. This study was carried out only in Thai subjects, a study in other ethnic population may provide variable evidence causes by ethnic-specific genetic variations as well as the different environmental factors. In addition, relatively limited sample sizes could affect the statistical power on detecting associations. Thus, these findings should be considered exploratory until they can be replicated in a larger sample size.

## Conclusions

In conclusion, we have demonstrated that four polymorphisms in antioxidant genes (*SOD2* rs4880*, SOD3* rs2536512 and rs2855262, and *GPX3* rs3828599) are independently associated with combined HTG and low HDL-C in subjects with high risk of CAD. We also provide evidence that high TG/HDL-C ratio predicts the severity of coronary atherosclerosis. We further demonstrated that high GRS derived from combined *SOD2*, *SOD3*, *GPX3,* and *GSTT1* polymorphisms have a strong association with such atherogenic dyslipidemia. Our finding provides the evidence of indirect linking between antioxidant gene variants and the severity of coronary atherosclerosis.

##  Supplemental Information

10.7717/peerj.7407/supp-1 Supplemental Information 1 Additional materials and methods (including genotyping of antioxidant gene polymorphisms and sample size calculation)

10.7717/peerj.7407/supp-2 Supplemental Information 2 Examples of DNA sequencing profiles

10.7717/peerj.7407/supp-3 Supplemental Information 3 Estimation of statistical power in high-risk group for the present study

10.7717/peerj.7407/supp-4 Supplemental Information 4 Additional results (including Figs. S1–S3 and Tables S3–S4)

10.7717/peerj.7407/supp-5 Supplemental Information 5 The raw measurements used for statistical analysis to evaluate the associations of individual and combined *SOD2*, *SOD3*, *GPX3*, and *GSTT1* gene polymorphisms with lipid abnormalities in subjects with high risk of CAD

## Additional Information and Declarations

### Data Availability

The following information was supplied regarding data availability: The raw measurements are available in the Supplemental File.
