# Supplementary material for: Association of genetic polymorphisms in SOD2, SOD3, GPX3, and GSTT1 with hypertriglyceridemia and low HDL-C level in subjects with high risk of coronary artery disease
Source: PeerJ. 2019 Aug 1;7:e7407. doi: 10.7717/peerj.7407 (PMC6679910; doi:10.7717/peerj.7407)
Supplement: Supplemental Information 1 [file peerj-07-7407-s001.docx]

**Supplementary Data 1**

**Materials and methods**

**Genotyping of antioxidant gene polymorphisms**

The primer sequences for *GSTT1* genotyping were followed Sprenger et al (Sprenger et al. 2000). Genotyping was performed in a final 25 μL PCR reaction buffer containing 100 ng genomic DNA, 1.0 µM of each primer, 2X PCR enhancer (Invitrogen), 1.5 mM MgCl_2_, 0.20 mM of each dNTP, and 0.5 unit of Taq DNA polymerase (Invitrogen). PCR reaction was performed with a 94°C initial denaturation step for 4.30 minutes, then followed by 30 cycles of [94°C denaturation (30 second), 65°C annealing (30 second), and 72°C extension reaction (90 second)], and a final step of 72°C for 7 minutes. The amplified products were size-separated on 2.0% agarose gel electrophoresis. Genotyping was based on size-specific PCR bands. The PCR product of 1,460 bps was interpreted as *GSTT1* null (-) allele, whereas the 466 bps product indicated *GSTT1* presented (+) allele.

The PCR primers for *GPX3* c.87+1494A>G (rs3828599) were 5’- TCCCCAACTCA GAAGGCATTTTCCA-3’ (outer forward primer), 5’-GGCATGCCCAGGCTTTCATTAGC-3’ (outer reverse primer), 5’-AGTCAGTCCCAACCTTCAGTTTTGGTAG-3’ (allele specific forward primer), and 5’-GCCCAATTGTATCTTCTTTGATCT-3’ (allele specific reverse primer). PCR was performed in a total 25 μL reaction buffer, consisting of 100 ng genomic DNA, 0.6 µM of each primer, 2.00 mM MgCl_2_, 0.20 mM of each dNTP, and 0.5 unit of Taq DNA polymerase (Invitrogen). The reaction cycle was 94°C initial denaturation step for 4.30 minutes, followed by 30 cycles of [94°C denaturation (30 second), 52°C annealing (30 second), and 72°C extension reaction (30 second)], and a final step of 72°C for 5 minutes. Genotyping was also based on the size-specific PCR bands as: AA (347 and 143 bps), AG (347, 143 and 255 bps), and GG (347 and 255 bps).

The primers for *SOD2* c.47C>T (rs4880) genotyping were 5’-GTGTGCGGG TGAGAAGAAAGG-3’ (outer forward primer), 5’-ACTTCTCCTCGGTGACGTTCAGGT-3’ (outer reverse primer), 5’-AGCAGGCAGCTGGCTCCAGT-3’ (allele specific forward primer), and 5’-TGGAGCCCAGATACCCCATAG-3’ (allele specific reverse primer). The PCR reaction was carried out as described above, except for using 1X PCR enhancer and 1.50 mM MgCl_2_, with 58^o^C annealing temperature. The genotypes were described according to size-specific PCR bands as: TT (471 and 178 bps), TC (471, 333 and 178 bps), and CC (471 and 333 bps).

The primers for *SOD3* c.172G>A (rs2536512) genotyping were 5’-TCCCGCA GGTGCCCGACTCCAG-3’ (outer forward primer), 5’-GGCGCAAGCTGCCGGAAGAGGA-3’ (outer reverse primer), 5’-CAGCGGCGGGACGACGACGACG-3’ (allele specific forward primer), and 5’- CTGGCAGGCGGCGTGGAGAGT-3’ (allele specific reverse primer). The PCR reaction was carried out as described above, except for using 1.0 µM of each primer, 1X PCR enhancer, and 1.75 mM MgCl_2_, with 63^o^C annealing temperature. The genotypes were described according to size-specific PCR bands as: GG (290 and 116 bps), GA (290, 116 and 216 bps), and AA (290 and 216 bps).

The primers for *SOD3* g.9892T>C (rs2855262) genotyping were 5’-GACGGCA GCCTCTGGAGGTA-3’ (outer forward primer), 5’-TCGGTACAAATGGAGGCCTTCAGA-3’ (outer reverse primer), 5’- TCCACTCTGAGGTCTCACCTTCGCGTT-3’ (allele specific forward primer), and 5’- AGGGCTGCGGGGAGACTTCAGGAG-3’ (allele specific reverse primer). The PCR reaction was carried out as described above except for 0.5 µM of each primer, 1X PCR enhancer, and 1.5 mM MgCl_2_, with 60^o^C annealing temperature and 55 second for 72°C extension reaction. The combination of size-specific PCR products indicating genotypes were 511 and 161 bps for TT; 511, 161 and 400 bps for TC; and 511 and 400 bps for CC.

**Sample size calculation**

Sample size calculation was performed before study completion, using statistical power of 80% and a significance level alpha of 0.05. Calculation was determined at 46.0% and 28.0% expected prevalence of *GSTT1*−/− in CAD cases and controls, respectively, from previous report (Charan and Biswas 2013; Manfredi et al. 2009) and found to consist of at least 121 subjects per group. Estimation of statistical power was performed using CaTS power calculator: (<http://www.sph.umich.edu/csg/abecasis/CaTS>) (as shown in Table S2, supplementary data 3).

**Table S1** Comparison of malondialdehyde (MDA) among genotypes in each polymorphism [243 pilot community-based subjects (aged >50 years) were recruited from Khon Kaen, Thailand]

| **Parameter** | ***SOD3***  **rs2536512** | | ***SOD3***  **rs2855262** | | ***GPX3***  **rs3828599** | | ***GSTT1* gene deletion polymorphism** | | ***SOD2***  **rs4880** | |
| --- | --- | --- | --- | --- | --- | --- | --- | --- | --- | --- |
| **Total subjects (n=243)** | | |  | |  | |  | |  |  |
| MDA (μmol/L) | GG (n=85) | 5.37±1.89 | TT (n=71) | 5.11±1.48 | GG (n=93) | 5.34±1.66 | +/+ (n=46) | 5.30±1.54 | TT (n=131) | 5.49±2.05 |
|  | GA (n=130) | 5.59±2.13 | TC (n=122) | 5.57±2.16 | GA (n=104) | 5.58±2.22 | +/- (n=116) | 5.62±2.27 | TC (n=96) | 5.56±1.96 |
|  | AA (n=28) | 5.51±1.80 | CC (n=50) | 5.91±2.20 | AA (n=46) | 5.66±2.15 | -/- (n=81) | 5.46±1.85 | CC (n=16) | 5.26±2.03 |
|  | *p*-value | 0.681 | *p*-value | 0.067 | *p*-value | 0.686 | *p*-value | 0.745 | *p*-value | 0.765 |
|  | *p* for trend | 0.624 | *p* for trend | **0.022** | *p* for trend | 0.412 | *p* for trend | 0.766 | *p* for trend | 0.564 |
| **Non-MetS subjects (n=149)** | | |  | |  | |  | |  |  |
| MDA (μmol/L) | GG (n=47) | 4.98±1.54 | TT (n=39) | 4.89±1.56 | GG (n=54) | 4.95±1.50 | +/+ (n=21) | 4.50±0.95 | TT (n=85) | 5.21±2.09 |
|  | GA (n=84) | 5.28±2.18 | TC (n=81) | 5.29±2.23 | GA (n=66) | 5.19±2.29 | +/- (n=77) | 5.40±2.25 | TC (n=55) | 5.02±1.52 |
|  | AA (n=18) | 4.81±0.92 | CC (n=29) | 4.99±1.04 | AA (n=29) | 5.30±1.47 | -/- (n=51) | 4.97±1.45 | CC (n=9) | 4.98±1.89 |
|  | *p*-value | 0.626 | *p*-value | 0.576 | *p*-value | 0.599 | *p*-value | 0.101 | *p*-value | 0.829 |
|  | *p* for trend | 0.894 | *p* for trend | 0.558 | *p* for trend | 0.313 | *p* for trend | 0.274 | *p* for trend | 0.628 |
| **MetS subjects (n=94)** | | |  | |  | |  | |  |  |
| MDA (μmol/L) | GG (n=38) | 5.84±2.17 | TT (n=32) | 5.37±1.36^a^ | GG (n=39) | 5.88±1.73 | +/+ (n=25) | 5.96±1.63 | TT (n=46) | 6.02±1.88 |
|  | GA (n=46) | 6.18±1.93 | TC (n=41) | 6.13±1.93 | GA (n=38) | 6.26±1.95 | +/- (n=39) | 6.06±2.27 | TC (n=41) | 6.29±2.24 |
|  | AA (n=10) | 6.79±2.30 | CC (n=21) | 7.17±2.72^a^ | AA (n=17) | 6.29±2.93 | -/- (n=30) | 6.28±2.16 | CC (n=7) | 5.63±2.29 |
|  | *p*-value | 0.350 | *p*-value | **0.009** | *p*-value | 0.704 | *p*-value | 0.875 | *p*-value | 0.663 |
|  | *p* for trend | 0.179 | *p* for trend | **0.002** | *p* for trend | 0.736 | *p* for trend | 0.705 | *p* for trend | 0.557 |

**Note:** Thiobarbituric acid (TBA) assay was used for the measurement of serum malondialdehyde (MDA) as an end product of lipid peroxidation (Nielsen et al. 1997; Wong et al. 1987). ^a^ *p*-value=0.010

**References**

**Charan J, Biswas T**. **2013**. How to calculate sample size for different study designs in medical research? *Indian journal of psychological medicine* 35:121-126. doi:10.4103/0253-7176.116232

**Manfredi S, Calvi D, del Fiandra M, Botto N, Biagini A, Andreassi MG**. **2009**. Glutathione S-transferase T1- and M1-null genotypes and coronary artery disease risk in patients with Type 2 diabetes mellitus. *Pharmacogenomics* 10:29-34. doi:10.2217/14622416.10.1.29

**Nielsen F, Mikkelsen BB, Nielsen JB, Andersen HR, Grandjean P**. **1997**. Plasma malondialdehyde as biomarker for oxidative stress: reference interval and effects of life-style factors. *Clinical Chemistry* 43:1209-1214

**Sprenger R, Schlagenhaufer R, Kerb R, Bruhn C, Brockmoller J, Roots I, Brinkmann U**. **2000**. Characterization of the glutathione S-transferase GSTT1 deletion: discrimination of all genotypes by polymerase chain reaction indicates a trimodular genotype-phenotype correlation. *Pharmacogenetics* 10:557-565

**Wong SH, Knight JA, Hopfer SM, Zaharia O, Leach CN, Jr., Sunderman FW, Jr. 1987**. Lipoperoxides in plasma as measured by liquid-chromatographic separation of malondialdehyde-thiobarbituric acid adduct. *Clinical Chemistry* 33:214-220
