# Supplementary material for: Association of genetic polymorphisms in SOD2, SOD3, GPX3, and GSTT1 with hypertriglyceridemia and low HDL-C level in subjects with high risk of coronary artery disease
Source: PeerJ. 2019 Aug 1;7:e7407. doi: 10.7717/peerj.7407 (PMC6679910; doi:10.7717/peerj.7407)
Supplement: Supplemental Information 2 [file peerj-07-7407-s002.pdf]

Supplementary Data 2

Examples of DNA sequencing profiles

1. *SOD2* c.47C>T (Ala16Val, rs4880)

SOD2 Ala16Val rs4880 (Ala: GCT→Val: GTT) (NG_008729.1:g.5482T>C)
CAGCACCAGCAGGCAGCTGGCTCCGG[C/T]TTTGGGGTATCTGGGCTCCAGGCAG

Example: Sequencing results

CAD011: TT

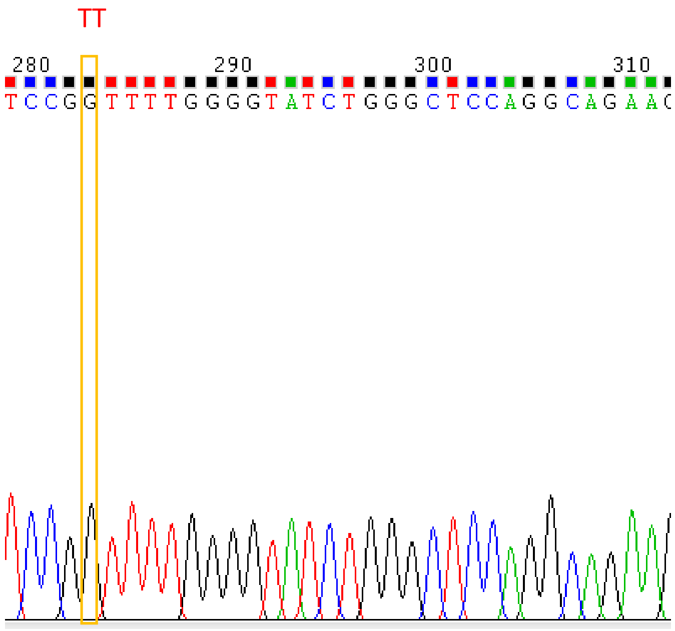

CAD068: CC

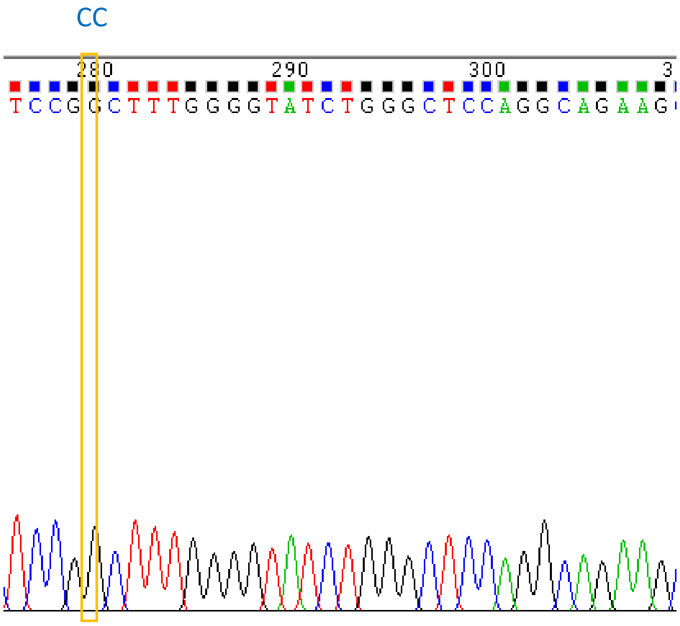

CAD147: TC

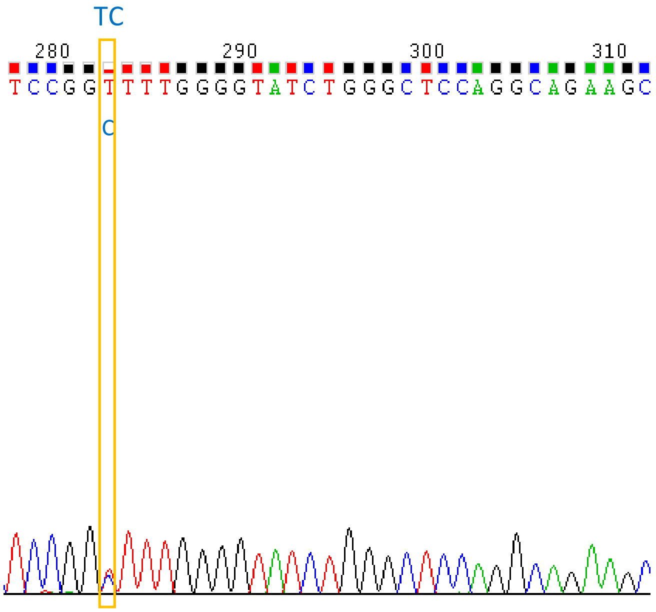

CADA030: TT

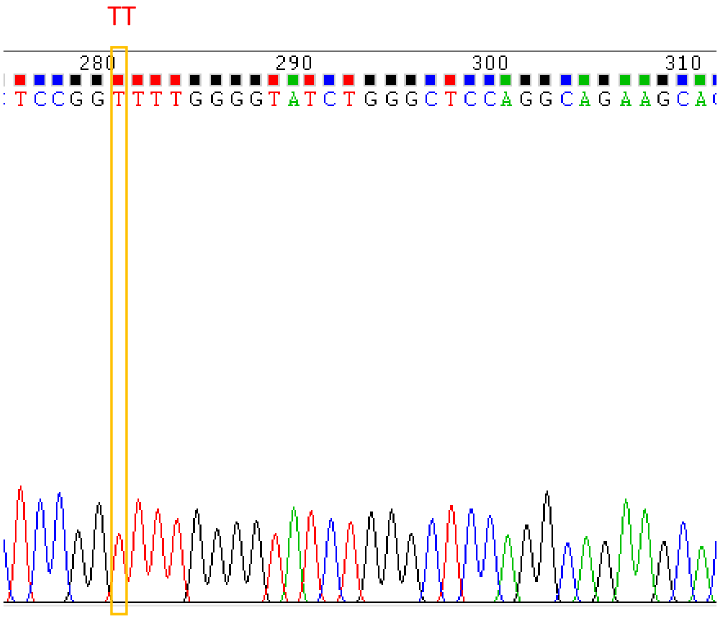

CADA121: TC

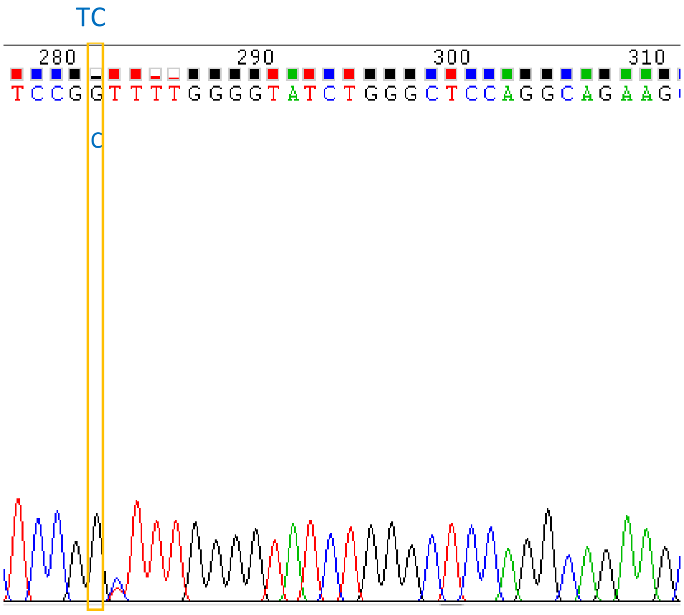

CADA128: CC

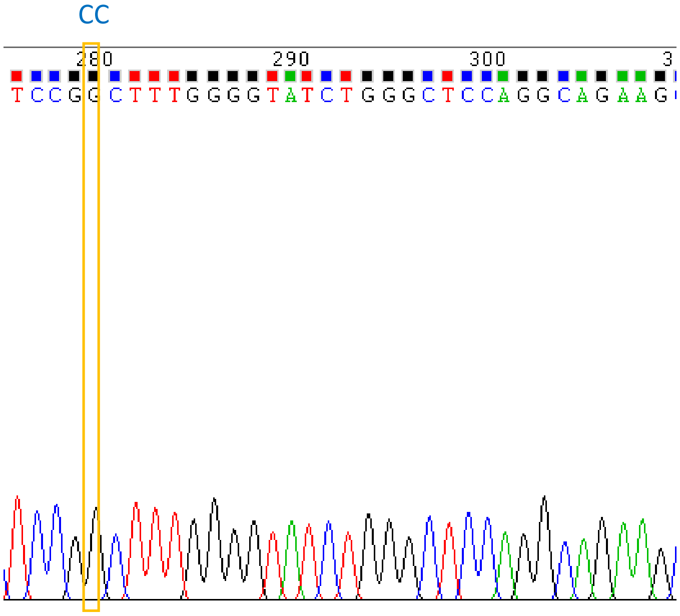

2. *SOD3* c.172G>A (Ala58Thr, rs2536512)

SOD3 Ala58Thr rs2536512 (Ala: GCG → Thr: ACG) NG_012213.1:g.9231G>A
TCATGCAGCGGCGGGACGACGACGGC[A/G]CGCTCCACGCCGCCTGCCAGGTGCA

Example: Sequencing results

CAD035: GG

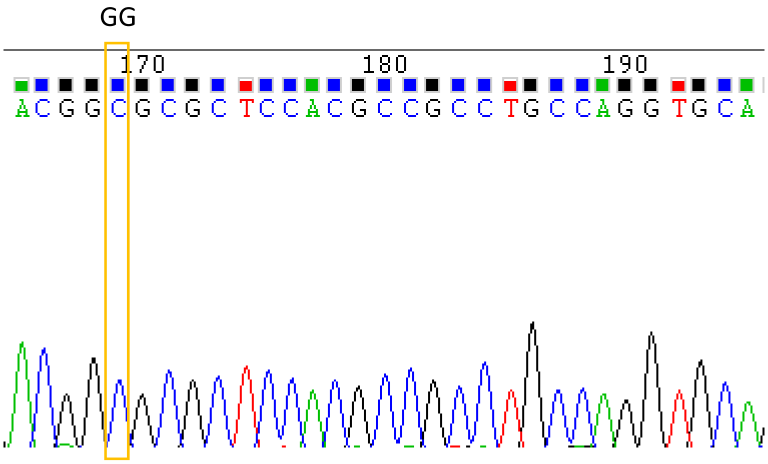

CAD046: GA

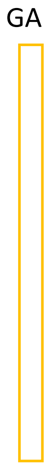

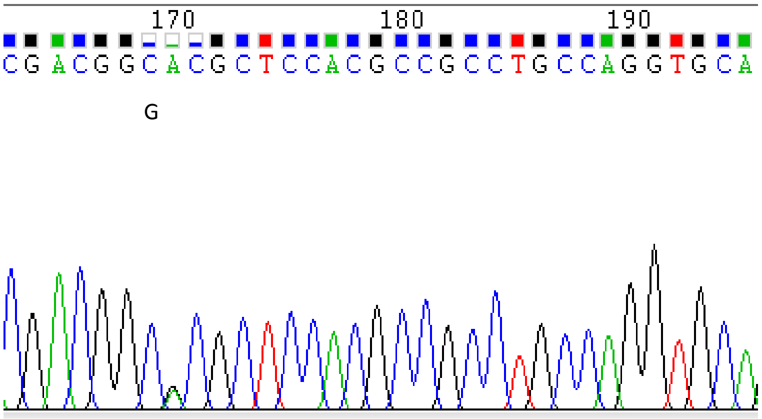

CADA017: GG

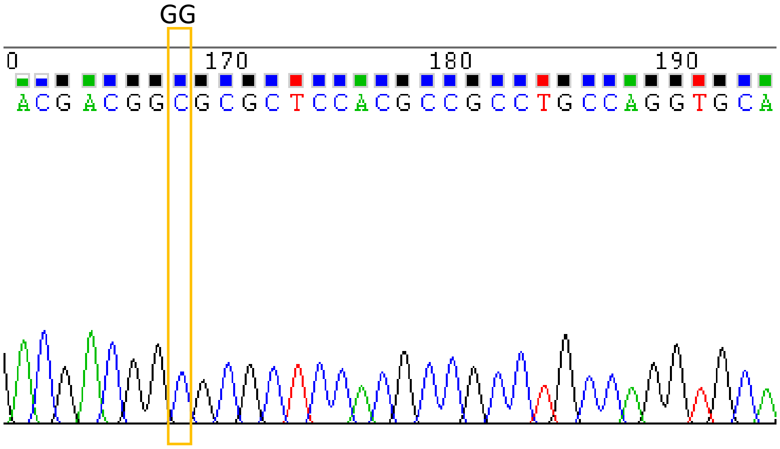

CADA088: GA

GA

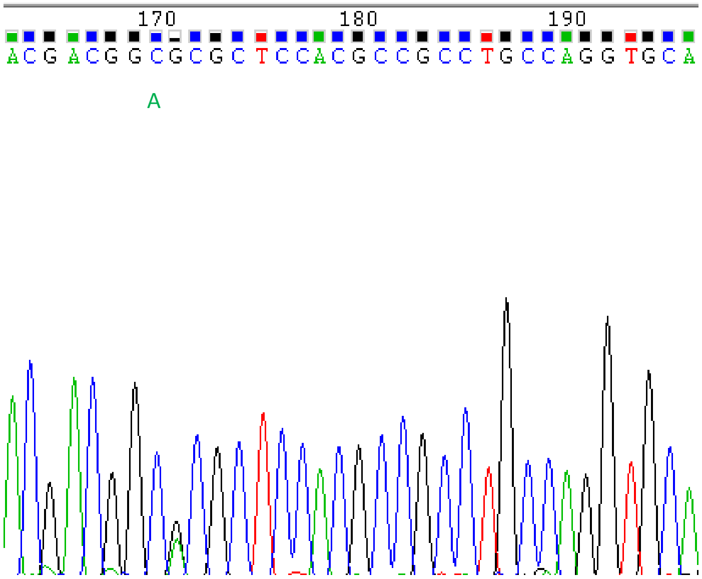

CADA092: AA

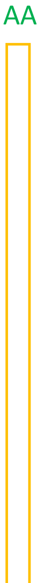

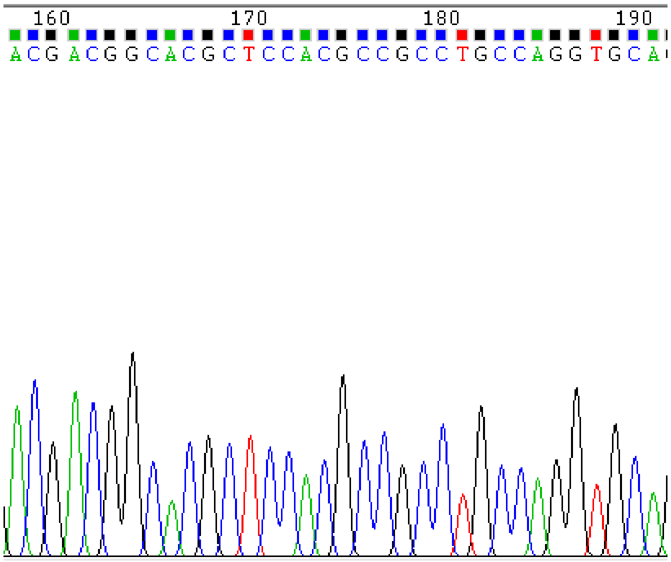

CADB025: AA

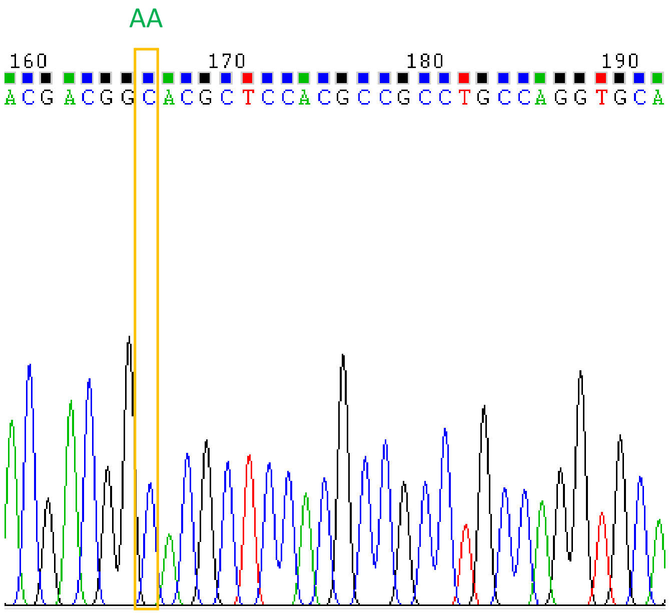

3. ***SOD3* g.9892T>C (rs2855262)**

SOD3 rs2855262 [NC_000004.11:g.24801976T>C] [NG_012213.1:g.9892T>C] [NM_003102.2:c.*110T>C] [NT_006316.16:g.15983773T>C] [NW_001838900.1:g.15423814C>C]
TCCACTCTGAGGTCTCACCTTCGCCT[C/T]TGCTGAAGTCTCCCCGCAGCCCTCT

Example: Sequencing results

CAD148: TC

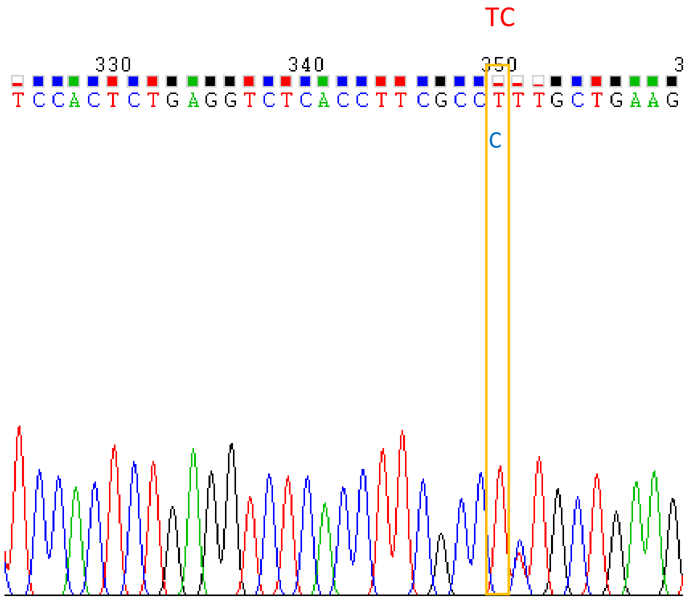

CADA050: TT

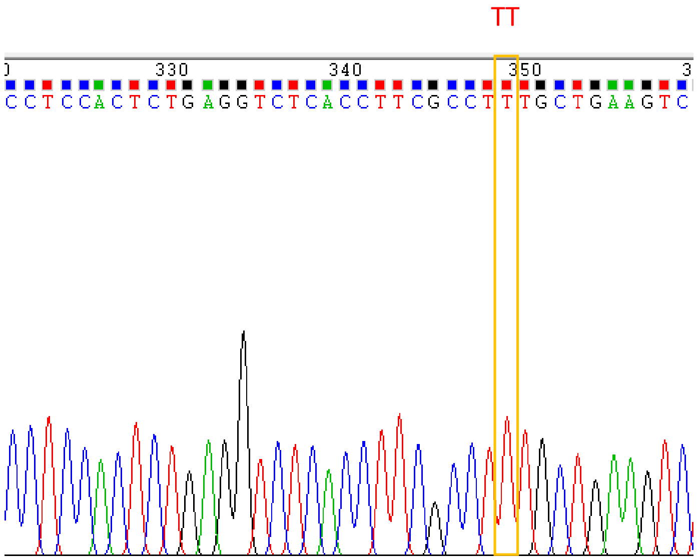

CADA069: TC

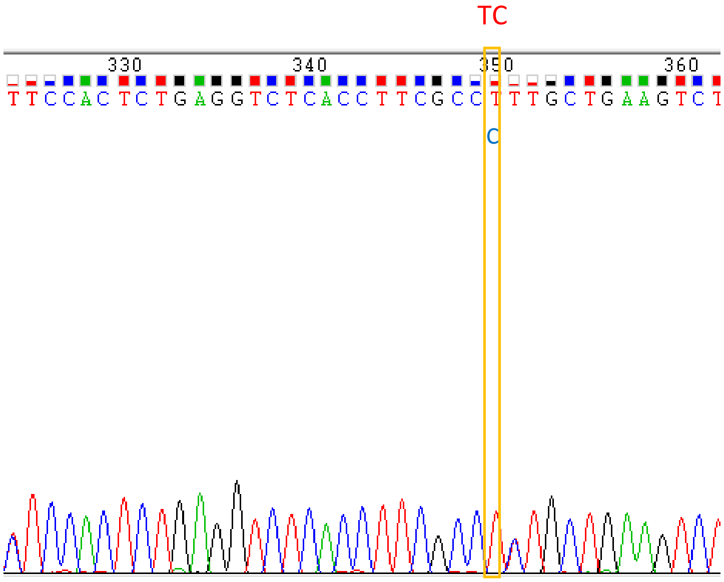

CADB016: CC

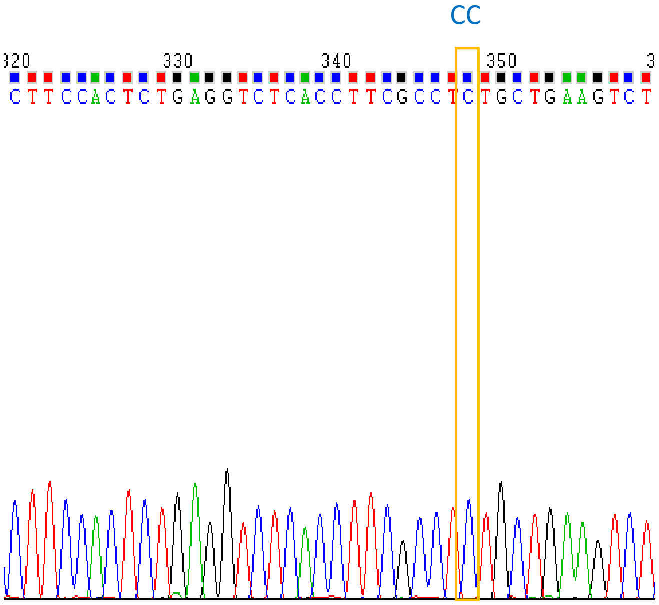

4. *GPX3* c.87+1494A>G (rs3828599)

GPX3 c.87+1494A>G [NC_000005.9:g.150401796A>G] [NM_002084.3:c.87+1494A>G]
[NT_029289.11:g.11564723A>G]

TCCCAACCTTCAGTTTTGGAA(G/A)GGTCAAAGAAGATACAATTGGGCCCTGC

Example: Sequencing results

CAD183: GG

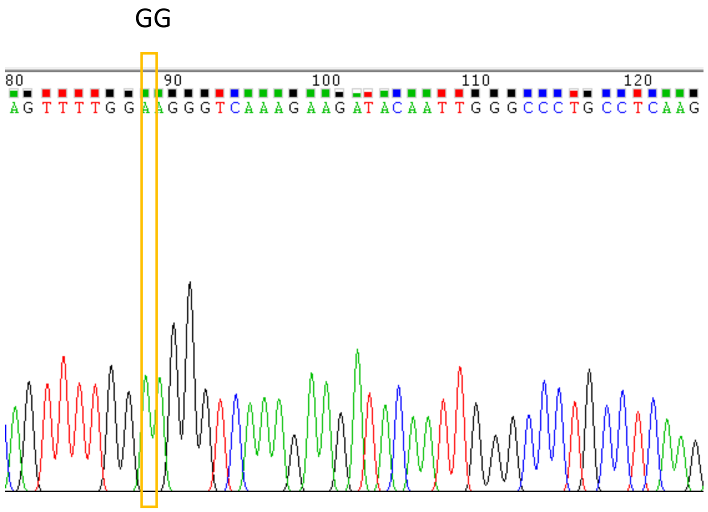

CAD120: GA

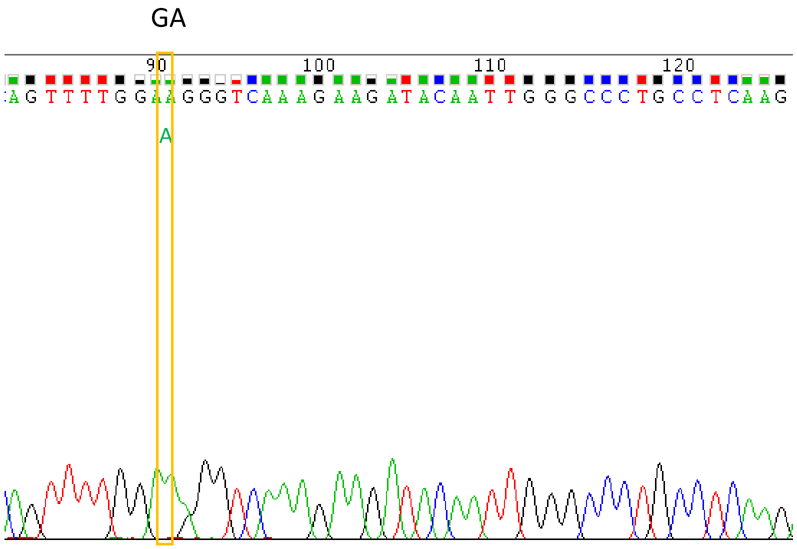

CAD155: GA

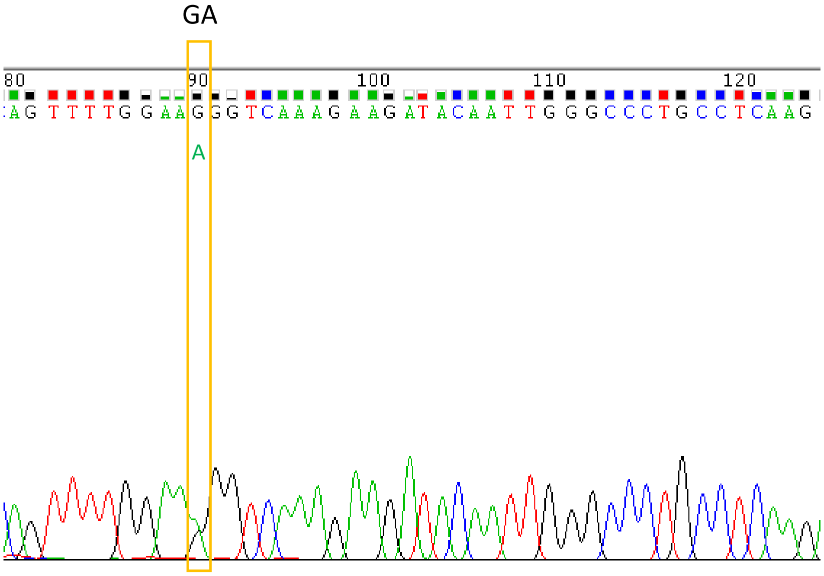

CADA094: AA

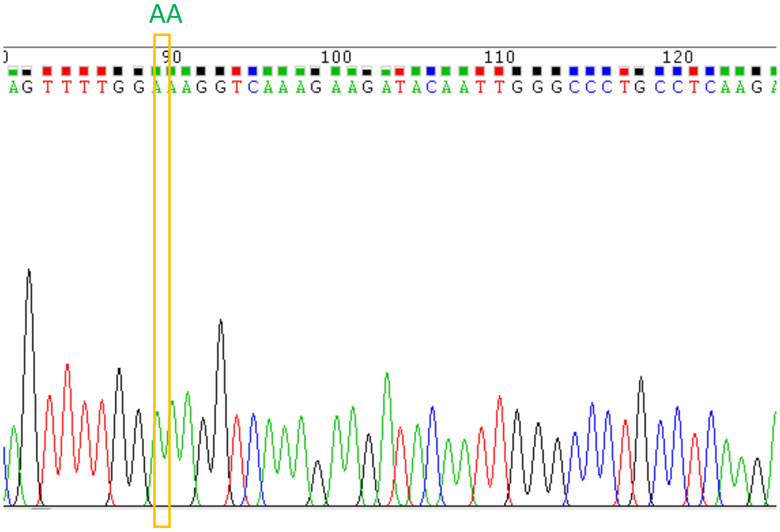

5. *GSTT1* deletion polymorphism

GSTT1 + genotype

GenBank: AF240786.1 (minus strand)

```
85081 AAGCTGACCC ACACGGCCTG GGAAGGGGGT TGTCTTTTGC ATAGAGACCA TGACCAGGTC
85141 TGGGACAGAG GAAAGTCAAA TAAATCACAC ATTAGAGTTA GAAGCAGAGG CTCAGGCTGA
85201 GCCCAGGTTT ATTATCCAAA ATCAAAATGA AATGCAGTGA TTAAAGGACA CAAGGCCTCA
85261 GTGTGCATCA TTCTCATTGT GGCTTTCAGG CGGCTGTGGA AGACAGGGTG GGGATGGTGG
85321 CTTCGGGAGG TGAGGTGCTC TGGGACTTGG GCAAGTCTTA GGCAAGCCAT TCCTGCTTTC
85381 TGGGCCTGGC TCCCATGGGC CATTAGAAAT GAAAATGCTT TGTGGACTGC TGAGGACGGT
      GSTT*A1 forward    CCAG CTCACCGGAT CATGGCCAG
85441 GCAAGGGTGA GGTTTCCCAG CTCACCGGAT CATGGCCAGC ACCCAGGGCA TCAGCTTCTG
85501 CTTTATGGTG GGGTCTGCAG GTGGGAAGTC CTTGGCCTTC AGAATGACCT CATGGGCCTC
85561 CTGGAAGAGG TCCTCCCCCA CTGCTGCCTC CACGCGCTGC CGCCATGTGG CCAGCTTGGG
85621 TCGGCCTTCG AAGACTTGGC AGCCAGCACC CACGGGCTGT GGGGAAAAGG GTACAGACTG
85681 GGGATGGATG GTTGTGAGGG CAGGGATGGG CAGCATCTGA TTTGGGGACC ACAGATCTCC
85741 AGGAGGTGTT TGCACACACA CTTAAGCACA GTGCCATAGC CCGGTGTGGC AGCATAAGCA
85801 GGACTTCAGC AACTAGCCAG GGCCCCCTGC CTAGTGGGTT CACCTGCCCA CAGCACTCAC
```

***GSTT1* exon 5 (minus strand)**

Example: Sequencing results

CAD146: +/+

GSTT1 + genotype

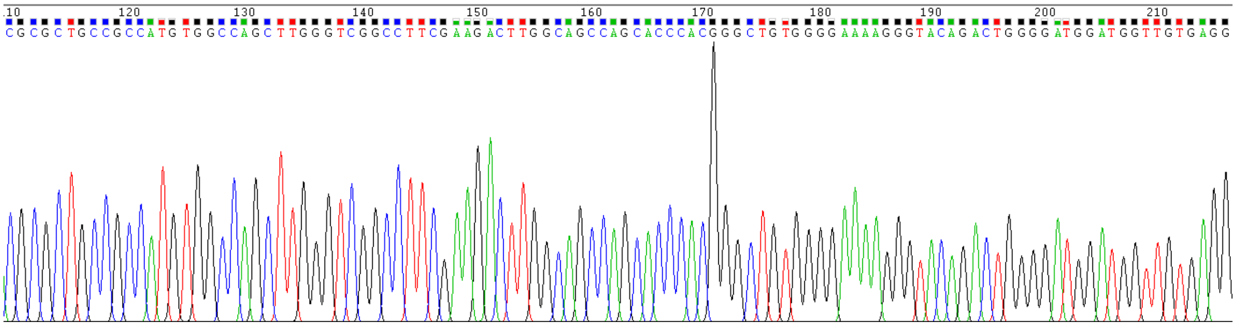

***GSTT1* - genotype**

GSTT*0 forward

```
6061 GGCCTCCCAA AGTACTGGGA TTACAGTTGT GAGCCACCGT ACCCGGCCTT CTGTGGGCCT
6121 TTTGACGCTG AGATTCTCTA GTTCAGAATG AAATGCCTCG AATGCTGTCC TGGGTGAGTC
6181 ACATCAGCCC CTCCCGTATG CCCTTCCCCT CGCCCTAATA AGACTCTTTC ATGCCCATTG
6241 TTTCAGTCCA CACTCCTGAC GGCTCTGTAA GGCGGGCAGG AGAAGGAGCT GAGGAAATGA
6301 GGCCCAGAGA GGGAGGGAGA CTTGCTTGAG GTCGCCGGCA GTCAGCAGGG CCAGAACTGG
6361 CCTCCAGCCT TCCTGACTTC CCTGTGCCCG TGTCCCCAAG CCCCAGAAGT GGCCCTGCTC
6421 ACCTTGAGCC AGACTATCTT CTTCATGGGC AGCTGAAAGG CCGTGTGTTG CCAAGCCACG
6481 AACTCATCCA CACGGGCACG TGCGTGCGGG TCTGGCGGGC ACCAGTGCGA TGGTGCGCTG
6541 TACTTGCGGC ACAGGTAGTA AAGGATGGCC GCGCTGCAGA AGGGGCCGGT CAGGGGCACT
6601 GCCCTTGCCT TCCTGAGTGC CACTACATCA ACCACCCCGG TGTGGCCTGG GCCCAACTGC
6661 TGGGGCTTCC AGAGCAAAGA GGAGCCCAAA CGGCCCCGAG AAAGACCTTC ACCAGAGCTG
6721 TCTGTCTGAC AGTCAGTAAG GGCTGGGAAG GAGCCCTGCG GGGTGAGTAG GAGTTGGGGG
6781 CTGGTGGTAT AACAAAGAGT AGGCCAGCAG GGGGAACAAC ACGTGTTGAA TTGGGATGCT
6841 GAGGTGGGAG GATCACTTGA TCCCAGGAAT TTGGGGCTAC TGTGAGCCAA GATCACACCA
6901 CTGCACTCCA GCTTGGGTGA AAGATCAAGA TCCTTTTTCA AAAACAAAAA CGGGGGGGCA
6961 CGATGGCTCA CACCTGTAAT CCCGGCACTT TGGGAGGCCA ATGGGGGCAG ATCCCTTGAG
7021 GCCAGGAGTT GGAGACCAGC CTGGCCAACA TGGTGAAACC CTGTCTCTAC TAAAATGAAA
7081 ATACAAAAAT TAGCTAGTTG TGGTGGCACA CACCTGTAAT CCCAGCTACT TGGGAAGCTG
7141 AGGCACGGGA GTCACTTGAA CCTGGGAGGC AGAGGTTGTA GTGAGCCAAG ATTGTGCCAC
7201 TGTACTCCAG CCTGGGCCAC AGAGCAAGAC TCTGTCTCAA AAAACCAACA AAGAAAAACA
7261 CATGCTGAAA TACGAGGGTA AAGGGAGCAA GGTAAATCTG AAGAAAAGAG AGTAGGGGGT
7321 TGCAACTGGA AGAAGGGTGG GGGTGATTGG GGAGTGATGA GGCAGCCAGA GACACTGTGG
7381 AGTCCACGGA GGGTAGCCCC TGGAGGTGCA GGGAGGTTAT GGACTTAATG CTTAAGATTA
7441 GGCATTATAT AAGCCAGGGC ATGAAAGGAT CCATCTCTCT GGTGCTGGAT GGAGGGTGAG
7501 CCCGAGGGGG CAGAATGGAC AATGAGGGGG CCAGCAACTA TCGGGAAGGT TGTGGTGTCT
                            CTCCCCC GGTCGTTGAT AGC GSTT*0 reverse
```

Example: Sequencing results

CADA033: -/-

GSTT1 - genotype

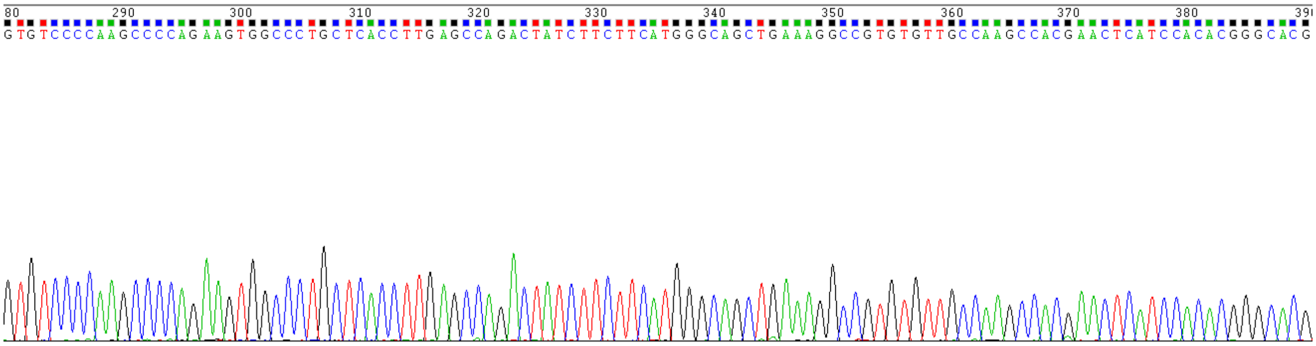
80 290 300 310 320 330 340 350 360 370 380 39
GTGTCCCCAAGCCCCAGAAGTGGCCCTGCTCACCTTGAGCCAGACTATCTTCTTCATGGGCAGCTGAAAGGCCGTGTGTTGCCAAGCCACGAACTCATCCACACGGGCACG
