# Supplementary material for: Association of genetic polymorphisms in SOD2, SOD3, GPX3, and GSTT1 with hypertriglyceridemia and low HDL-C level in subjects with high risk of coronary artery disease
Source: PeerJ. 2019 Aug 1;7:e7407. doi: 10.7717/peerj.7407 (PMC6679910; doi:10.7717/peerj.7407)
Supplement: Supplemental Information 4 [file peerj-07-7407-s004.docx]

**Supplementary Data 4**

**Results**

**
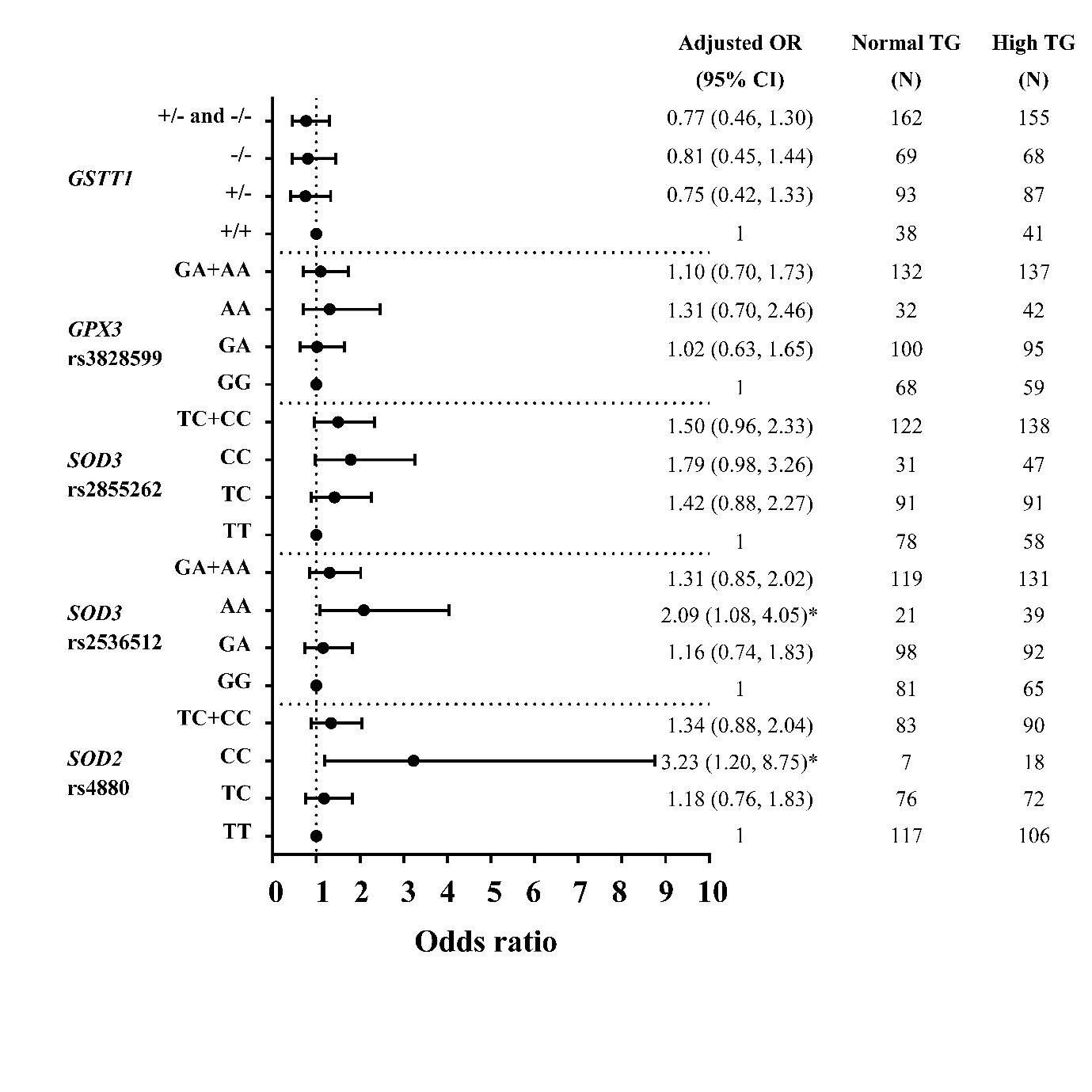
**

**Figure S1** Adjusted Odds ratios (ORs) and 95% CI for the risk of HTG as the function of antioxidant related gene polymorphisms. The ORs were adjusted for age, gender, prevalence of having low HDL-C level, smoking status, and administration of fibrate. N: total number of genotype carriers in each group**.** * indicates statistically significant; *p* <0.05.


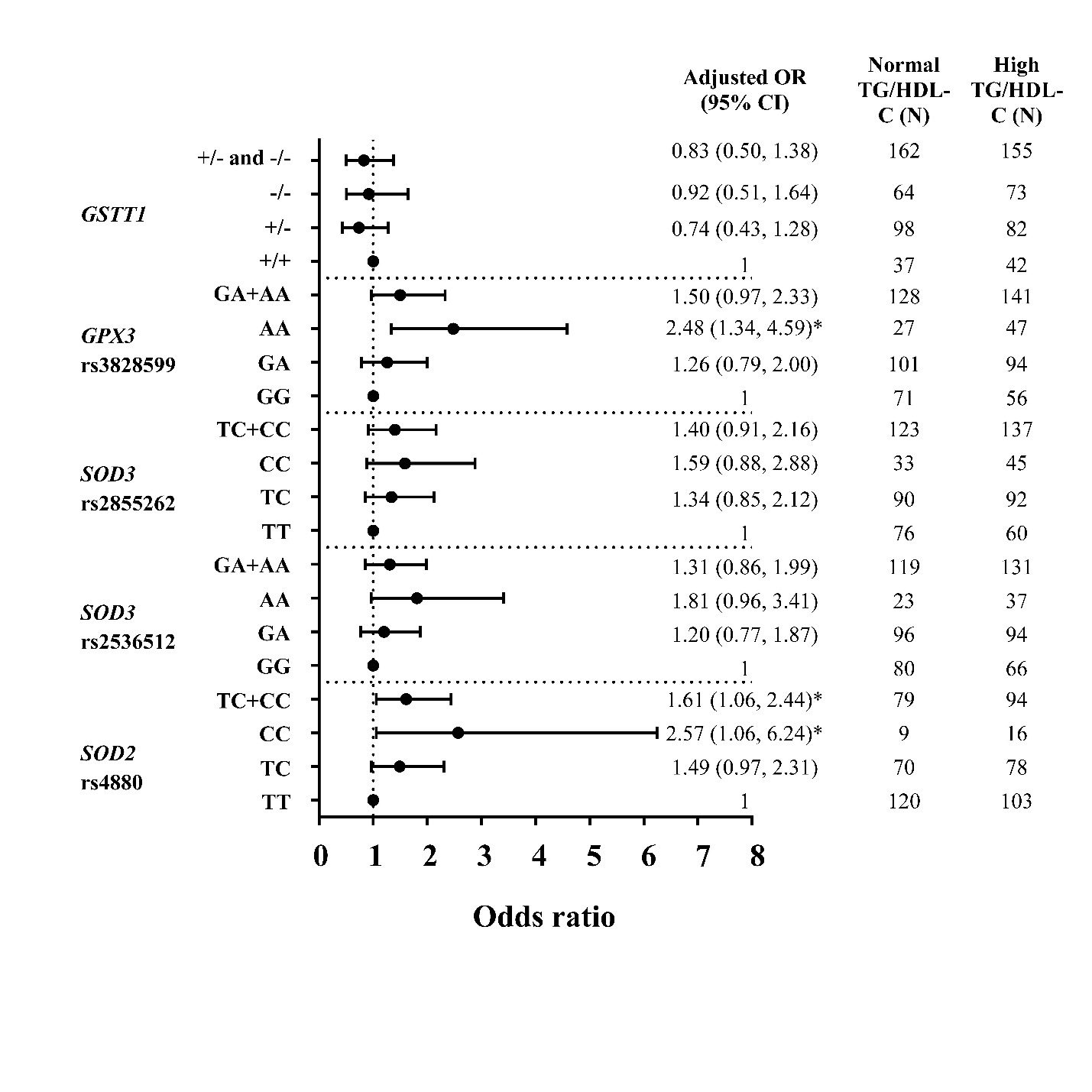


**Figure S2** Adjusted Odds ratios (ORs) and 95% CI for the risk of high TG/HDL-C ratio (≥3.7) as the function of antioxidant related gene polymorphisms. The ORs were adjusted for age, gender, smoking status, and administration of fibrate. N: total number of genotype carriers in each group**.** * indicates statistically significant; *p* <0.05.

**
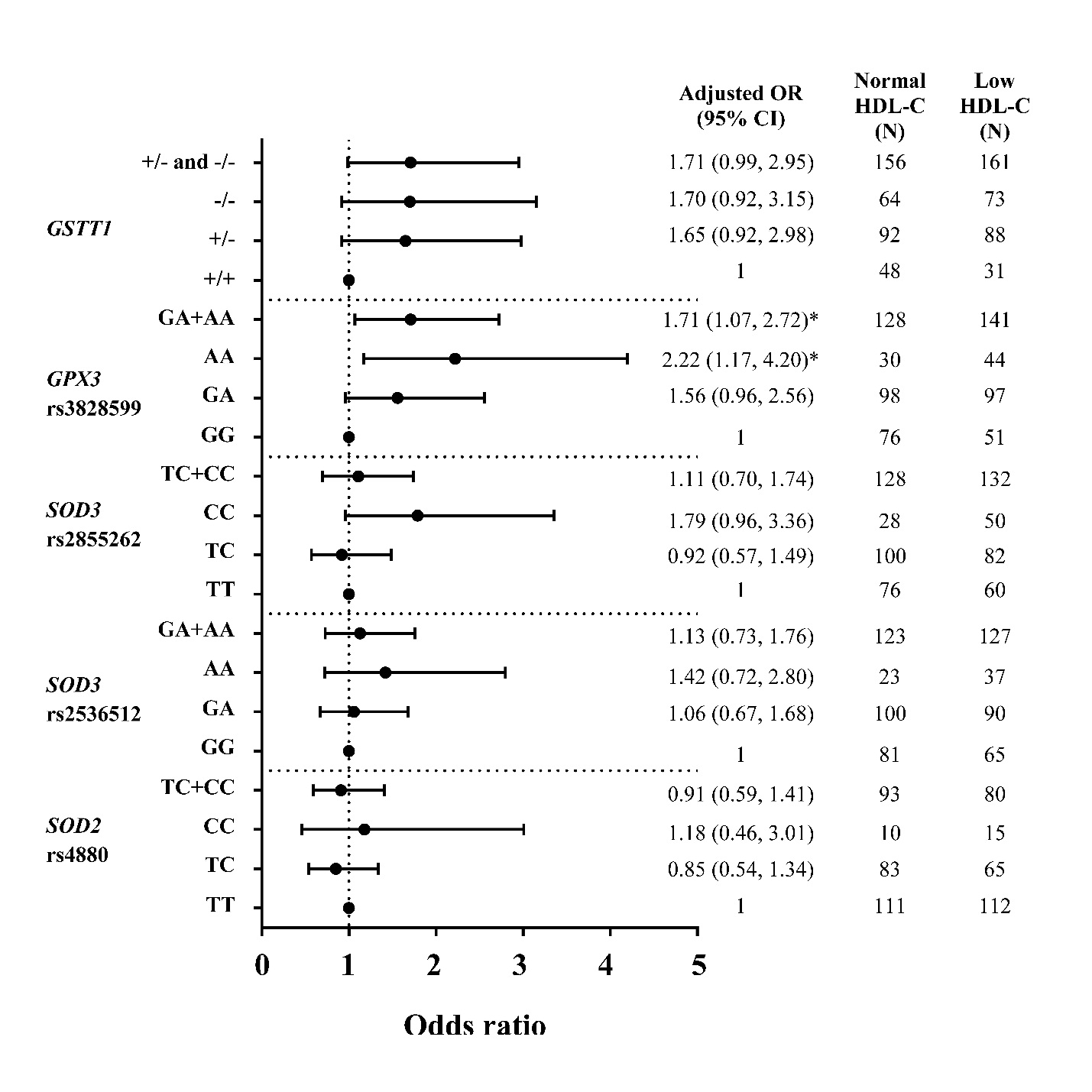
**

**Figure S3** Adjusted Odds ratios (ORs) and 95% CI for the risk of low HDL-C (<1 mmol/L) as the function of antioxidant related gene polymorphisms. The ORs were adjusted for age, gender, prevalence of HTG, smoking status, and administration of fibrate. N: total number of genotype carriers in each group**.** * indicates statistically significant; *p* <0.05.

**Table S3** The associations between antioxidant gene polymorphisms and the risk of Gensini score >32

| **Polymorphisms** | **Gensini**  **score**  **≤32; N (%)**  **(n=268)** | **Gensini score**  **>32; N (%)**  **(n=121)** | **Crude odds ratio (95%CI)** | ***p*-value** | **^a^Adjusted Odds Ratio**  **(95% CI)** | ***p*-value** |
| --- | --- | --- | --- | --- | --- | --- |
| ***SOD2* rs4880** |  |  |  |  |  |  |
| TT | 151 (56.3%) | 69 (57.0%) | 1 | - | 1 | - |
| TC | 102 (38.1%) | 42 (34.7%) | 0.90 (0.57, 1.43) | 0.656 | 0.95 (0.49, 1.82) | 0.866 |
| CC | 15 (5.6%) | 10 (8.3%) | 1.46 (0.62, 3.41) | 0.383 | 1.88 (0.49, 7.28) | 0.361 |
| TC+CC | 117 (43.7%) | 52 (43.0%) | 0.97 (0.63, 1.50) | 0.900 | 1.04 (0.56, 1.95) | 0.896 |
| ***SOD3* rs2536512** |  |  |  |  |  |  |
| GG | 106 (39.6%) | 37 (30.6%) | 1 | - | 1 | - |
| GA | 120 (44.8%) | 67 (55.4%) | 1.60 (0.99, 2.58) | 0.055 | 1.75 (0.84, 3.61) | 0.133 |
| AA | 42 (15.7%) | 17 (14.0%) | 1.16 (0.59, 2.28) | 0.668 | 1.54 (0.56, 4.26) | 0.407 |
| GA+AA | 162 (60.4%) | 84 (69.4%) | 1.49 (0.94, 2.35) | 0.090 | 1.73 (0.86, 3.47) | 0.123 |
| ***SOD3***  **rs2855262** |  |  |  |  |  |  |
| TT | 98 (36.6%) | 35 (28.9%) | 1 | - | 1 | - |
| TC | 118 (44.0%) | 61 (50.4%) | 1.45 (0.88, 2.37) | 0.143 | 1.58 (0.76, 3.30) | 0.219 |
| CC | 52 (19.4%) | 25 (20.7%) | 1.35 (0.73, 2.49) | 0.342 | 1.33 (0.51, 3.47) | 0.563 |
| TC+CC | 170 (63.4%) | 86 (71.1%) | 1.42 (0.89, 2.26) | 0.142 | 1.50 (0.75, 3.02) | 0.254 |
| ***GPX3* rs3828599** |  |  |  |  |  |  |
| GG | 80 (29.9%) | 43 (35.5%) | 1 | - | 1 | - |
| GA | 137 (51.1%) | 56 (46.3%) | 0.76 (0.47, 1.23) | 0.267 | 1.08 (0.54, 2.17) | 0.822 |
| AA | 51 (19.0%) | 22 (18.2%) | 0.80 (0.43, 1.50) | 0.488 | 1.46 (0.53, 4.03) | 0.469 |
| GA+AA | 188 (70.1%) | 78 (64.5%) | 0.77 (0.49, 1.22) | 0.265 | 1.15 (0.59, 2.24) | 0.673 |
| ***GSTT1* gene deletion polymorphism** |  |  |  |  |  |  |
| +/+ | 56 (20.9%) | 19 (15.7%) | 1 | - | 1 | - |
| +/- | 121 (45.1%) | 57 (47.1%) | 1.39 (0.76, 2.55) | 0.290 | 1.12 (0.43, 2.92) | 0.823 |
| -/- | 91 (34.0%) | 45 (37.2%) | 1.46 (0.78, 2.74) | 0.242 | 1.96 (0.66, 5.83) | 0.228 |
| +/- and -/- | 212 (79.1%) | 102 (84.3%) | 1.42 (0.80, 2.51) | 0.231 | 1.50 (0.61, 3.69) | 0.381 |

**Note:** ^a^Data was calculated with logistic regression analysis adjusted for age, gender, DM, HT, waist/hip ratio, and cigarette smoking.

**Table S4** The associations between genetic risk score and the risk of Gensini score >32

| **Genetic risk score** | **Gensini**  **score**  **≤32; N (%)**  **(n=268)** | **Gensini score**  **>32; N (%)**  **(n=121)** | **Crude odds ratio (95%CI)** | ***p*-value** | **^a^Adjusted Odds Ratio**  **(95% CI)** | ***p*-value** |
| --- | --- | --- | --- | --- | --- | --- |
| **0-2** | 52 (19.4%) | 19 (15.7%) | 1 | - | 1 | - |
| **3** | 56 (20.9%) | 23 (19.0%) | 1.12 (0.55, 2.30) | 0.749 | 1.20 (0.38, 3.81) | 0.757 |
| **4-5** | 100 (37.3%) | 53 (43.8%) | 1.45 (0.78, 2.70) | 0.241 | 1.77 (0.67, 4.70) | 0.250 |
| **6-10** | 60 (22.4%) | 26 (21.5%) | 1.19 (0.59, 2.38) | 0.632 | 2.32 (0.66, 8.13) | 0.187 |

**Note:** ^a^Data was calculated with logistic regression analysis adjusted for age, gender, DM, HT, waist/hip ratio, and cigarette smoking.
